# Tumour-targeted interleukin-12 and entinostat combination therapy improves cancer survival by reprogramming the tumour immune cell landscape

**DOI:** 10.1038/s41467-021-25393-x

**Published:** 2021-08-26

**Authors:** Kristin C. Hicks, Paul L. Chariou, Yohei Ozawa, Christine M. Minnar, Karin M. Knudson, Thomas J. Meyer, Jing Bian, Margaret Cam, Jeffrey Schlom, Sofia R. Gameiro

**Affiliations:** 1 Laboratory of Tumor Immunology and Biology, Center for Cancer Research, National Cancer Institute, National Institutes of Health, Bethesda, MD USA; 2 CCR Collaborative Bioinformatics Resource (CCBR), Center for Cancer Research, National Cancer Institute, National Institutes of Health, Bethesda, MD USA

**Keywords:** Cancer microenvironment, Immunotherapy, Immunoediting, Tumour vaccines

## Abstract

Poorly inflamed carcinomas do not respond well to immune checkpoint blockade. Converting the tumour microenvironment into a functionally inflamed immune hub would extend the clinical benefit of immune therapy to a larger proportion of cancer patients. Here we show, by using comprehensive single-cell transcriptome, proteome, and immune cell analysis, that Entinostat, a class I histone deacetylase inhibitor, facilitates accumulation of the necrosis-targeted recombinant murine immune-cytokine, NHS-rmIL12, in experimental mouse colon carcinomas and poorly immunogenic breast tumours. This combination therapy reprograms the tumour innate and adaptive immune milieu to an inflamed landscape, where the concerted action of highly functional CD8^+^ T cells and activated neutrophils drive macrophage M1-like polarization, leading to complete tumour eradication in 41.7%-100% of cases. Biomarker signature of favourable overall survival in multiple human tumor types shows close resemblance to the immune pattern generated by Entinostat/NHS-rmIL12 combination therapy. Collectively, these findings provide a rationale for combining NHS-IL12 with Entinostat in the clinical setting.

## Abstract (web summary)

The immunocytokine NHS-IL12 is targeted to necrotic tumour areas to prompt an immune response. Authors show here in mouse colon and breast models that the combination of histone deacetylase inhibitor, Entinostat, and NHS-IL12 generates a tumour microenvironment that favours tumour resolution.

## Introduction

Immunotherapy targeting immune checkpoints achieved durable responses across multiple tumor types. However, clinical benefit is elusive for most patients with cancer, resulting in low response rates and lack of complete responses^1^. In 2020, approximately 600,000 people died of cancer in the U.S. alone^2^. Thus, in addition to the spectrum of checkpoint inhibitor monoclonal antibodies currently being employed as immunotherapeutics, immune-mediating agents and strategies need to be designed and evaluated. Effective immune-mediated attack of established poorly immunogenic carcinomas requires sufficient and highly functional immune effector cells in the tumor microenvironment (TME) able to efficiently sustain tumor destruction^3^. There is thus an unmet clinical need for therapeutic strategies to convert the TME to an effective functionally inflamed immune landscape able to promote and sustain significant clinical benefit.

Interleukin-12 (IL-12) is a cytokine for cancer immunotherapy providing a critical bridge between innate and adaptive immunity^4^. Mainly produced by activated antigen-presenting cells including dendritic cells (DCs), monocytes, and macrophages, IL-12 induces proliferation and lytic function of natural killer (NK), NKT, and T cells^5^. IL-12-driven polarization of T cells into a type 1 (Th1) effector phenotype stimulates cytokine secretion, notably IFNγ, favoring cell-mediated immunity^6^. IFNγ induces macrophage secretion of anti-angiogenic chemokines CXCL9/MIG (monokine induced by IFNγ) and CXCL10/IP-10 (interferon-inducible protein 10), both positive regulators of T-cell chemotaxis^7^. Direct effects on DCs further amplify IL-12 production while enhancing antigen presentation^8^.

Despite encouraging preclinical^9–11^ and some clinical^12–15^ data, a narrow therapeutic window with documented cases of severe toxicity observed in early trials with systemic administration of recombinant human IL-12 (rhIL-12) hindered further clinical development^4,16,17^. Hence, innovative strategies aimed at lessening toxicity induced by systemic exposure and improving response rates are highly desirable. Promising preclinical findings have been shown with intratumoral administration of collagen-binding IL-12 RNA nanoparticles^18^. However, whereas intratumoral IL-12 administration has been examined both preclinically^4,19,20^ and in clinical studies^21^, this constitutes a challenging approach for most solid malignancies. Recent preclinical studies have shown promising results with intravenous administration of collagen- and fibronectin-binding IL-12^22,23^. However, a strategy selectively targeting IL-12 to the TME and enabling subcutaneous (s.c.) administration may promote effective tumor control with manageable toxicity, while widely applicable to non-superficial and metastatic malignancies.

NHS-IL12 is a recombinant immunocytokine composed by the human antibody NHS76 fused to two IL12 heterodimers^24^. NHS76 targets exposed DNA in necrotic areas^9,25^, thus directing IL-12 to the TME, and reducing systemic exposure^9,11^. Preclinically, NHS-rmIL12 demonstrated superior antitumor activity to rmIL12^9^. In a first-in-human phase I trial in a wide range of locally advanced and metastatic malignancies^26^, NHS-IL12 was well tolerated and showed signs of clinical activity, albeit no objective responses.

Mounting preclinical evidence suggests that the antitumor potential of NHS-IL12 may be best realized in combination with other treatment modalities^9–11,27,28^. Preclinical and clinical evidence suggest that immunotherapy combined with epigenetic modulators, such as histone deacetylase inhibitors (HDACi), can attain significant antitumor efficacy^29–31^. Entinostat, a class I HDACi, inhibits regulatory T cells (Tregs) and myeloid-derived suppressor cells (MDSCs)^31–36^, and promotes tumor infiltration of lytic CD8^+^ T cells^31^. Moreover, Entinostat renders carcinoma cells more amenable to immune killing^37–39^, in part by upregulating major histocompatibility complex I and antigen presentation. The preclinical observation that Entinostat can increase tumor necrosis^31^ led us to hypothesize that immune-complementary effects of NHS-rmIL12 and Entinostat synergize to promote significant antitumor efficacy in well-established tumors.

Here, we show that combination therapy with Entinostat plus NHS-rmIL12 elicits significant survival benefit in murine models with a range of immunogenicity and sensitivity to PD-L1 blockade, namely poorly immunogenic EMT6 breast tumors, and MC38 and CT26 colon carcinoma models. Using comprehensive TME single-cell transcriptome, proteome, and immune cell analysis, we identify an immunologic cross-talk between innate and adaptive immunity conducive to the eradication of established tumors. Further, we identify a biomarker signature in the TME associated with patients’ overall survival across multiple tumor types.

## Results

### Tumor-targeted NHS-rmIL12 plus Entinostat eradicates well-established tumors in multiple murine carcinoma models

**Fig. 1:**
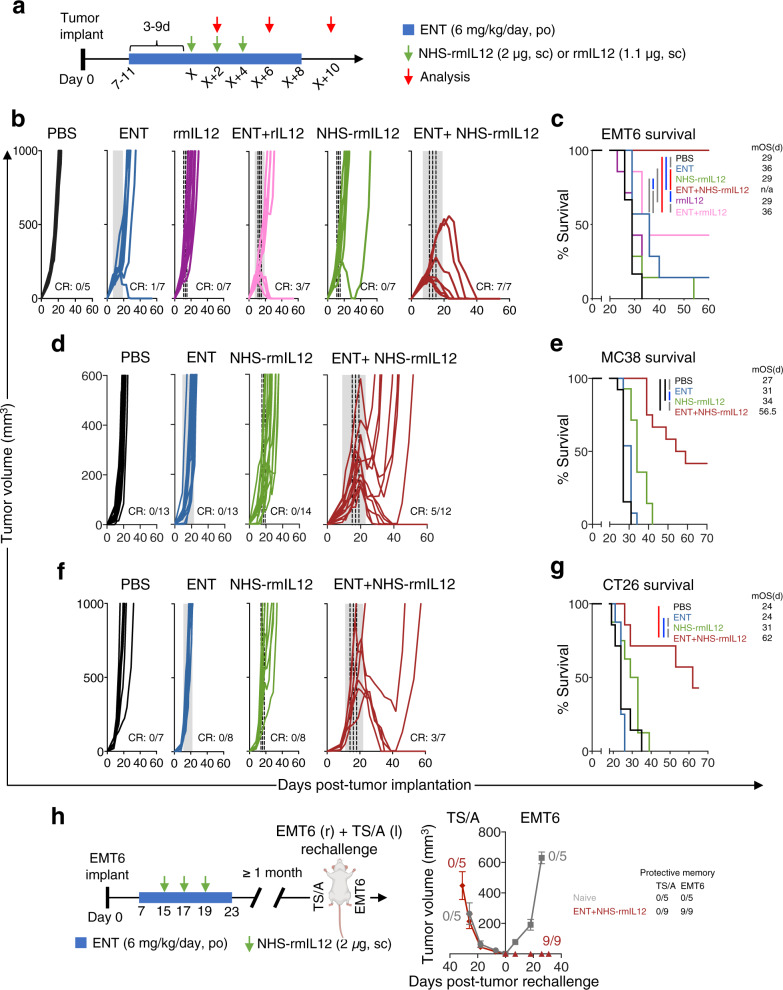
Combination therapy with Entinostat and NHS-rmIL12 eradicates established EMT6, MC38 and CT26 tumors. **a** Generic treatment schedule for all tumor models. Red arrows indicate days when analyses were performed. Green arrows denote days of NHS-rmIL12 or rmIL12 treatment and “X” denotes day of first dosing. Blue denotes duration of Entinostat (ENT) dosing. **b** Individual tumor growth curves of EMT6-tumor-bearing mice treated as depicted in (**a**). Gray shaded area indicates ENT treatment and dashed line depicts NHS-rmIL12 or rmIL12 dosing. CR, complete response. **c** Graph shows survival and table shows median overall survival (mOS), *P* < 0.0001. PBS vs. ENT+NHS-rmIL12, *P* = 0.0003; PBS vs. ENT+rmIL12, *P* = 0.006; ENT vs. ENT+NHS-rmIL12, *P* = 0.0013; NHS-rmIL12 vs. ENT+NHS-rmIL12, *P* = 0.0001. **d** Individual tumor growth curves and (**e**) survival (inset indicates mOS) for MC38-tumor-bearing mice treated as depicted in (**a**). For survival, *P* < 0.0001. PBS or ENT or NHS-rmIL12 vs. ENT+NHS-rmIL12, *P* < 0.0001. **f** Individual tumor growth curves and (**g**), survival (inset indicates mOS) of CT26 tumor-bearing mice treated as depicted in (**a**). For survival, *P* = 0.0004. PBS vs. ENT+NHS-rmIL12, *P* = 0.0030; ENT vs. ENT+NHS-rmIL12, *P* = 0.0005; NHS-rmIL12 vs. ENT+NHS-rmIL12, *P* = 0.0120. **h** Naïve (*n* = 5) and combination-treated mice previously cured from EMT6 tumors (*n* = 9) were implanted with EMT6 in the right mammary fat pad and TS/A in the opposing left as depicted in the schematic. Mouse icon adapted from BioRender.com. All other elements in the schematic were inserted from Microsoft Powerpoint for Mac v 16.49 (Microsoft). Graph indicates tumor growth on each side as mean ± SEM. Table denotes number of mice with protective memory. Data from one independent experiment, except for CT26 and EMT6 tumor growth and survival with ENT+NHS−rmIL12 treatment, which is representative of two and three experiments conducted independently with similar results, respectively. For survival analysis, two-tailed Log-rank (Mantel–Cox) was used for comparisons. Grey = *p* < 0.05, red = *p* < 0.01, blue = *p* < 0.001, black = *p* < 0.0001. Source data are provided as a Source Data file.

Preclinically, both NHS-rmIL12 and Entinostat have been shown to promote partial control of early stage tumors as single agents, with unremarkable efficacy against larger, more established tumors^9,10,31^. We hypothesized that combining these therapies would be efficacious against more advanced, larger breast and colorectal tumors. We employed three distinct carcinoma models in these studies. The EMT6 breast carcinoma is a poorly immunogenic tumor model that has been shown by us and others to have a poor to moderate response to anti-PD-L1 therapy^27,40^. CT26 and MC38 are immunogenic colon carcinoma models that are poorly and partially responsive to anti-PD-L1 therapy, respectively^27,40,41^. Entinostat was administered continuously in the diet to better mimic the longer half-life observed in cancer patients relative to mice^31,42,43^. Mice bearing EMT6 tumors were randomized to control or Entinostat diet once tumors reached 50–100 mm^3^ ranging 7–11 days after tumor implant (Fig. 1a). Three to nine days later, when tumors reached 150-200 mm^3^, mice received NHS-rmIL12 (2μg, s.c.) every other day for three doses. The Entinostat diet was removed 4 days after the last dose of NHS-rmIL12. Using this treatment schedule, the safety of the combination therapy was assessed. Entinostat-treated mice displayed transient weight loss that quickly reversed after Entinostat cessation. Whereas combination therapy induced mild, transient, albeit significant alterations in serum chemistry, baseline levels resumed post-treatment. No overt additional toxicity was observed by organ histopathology relative to other tumor-bearing mice (Supplementary Fig. 1).

To probe if targeting IL-12 to the TME was determinant to the antitumor efficacy of combination therapy, NHS-rmIL12 was compared to rmIL12, alone or in combination with Entinostat in the EMT6 breast carcinoma model. Both rmIL12 and NHS-rmIL12 showed negligible efficacy against the larger EMT6 tumors (Fig. 1b, c). In combination with Entinostat, rmIL12 significantly extended survival by 7 days versus PBS treatment. However, Entinostat combined with NHS-rmIL12 resolved all tumors with full survival benefit, indicating that targeting IL-12 to the TME is determinant to tumor resolution. Next, we examined the antitumor efficacy of the NHS-rmIL12 combination in two additional tumor models. In MC38 and CT26 colorectal cancer models, both Entinostat and NHS-rmIL12 monotherapies showed modest efficacy (Fig. 1d–g). However, their combination resulted in effective tumor regression with a resolution of 41.7% and 42.9% of MC38 and CT26 tumors, respectively (Fig. 1d, f). This led to a significant increase in survival of 30 and 38 days relative to PBS treatment in MC38 and CT26 models, respectively (Fig. 1e, g). Additional treatment schedules were also examined to determine if early termination or a lead-in of Entinostat treatment was the determinant of full tumor eradication. Whereas antitumor efficacy was not altered by continuous Entinostat administration, delaying its onset to the time of first NHS-rmIL12 dosing negated the survival benefit of combination therapy (Supplementary Fig. 2). Thus, all subsequent studies were performed with the initial treatment schedule (Fig. 1a).

We observed that all cured mice in all three tumor models displayed protective memory against tumor rechallenge (Supplementary Fig. 2). To probe if this effect was CD8-specific, cured mice were depleted of CD8^+^ T cells prior to, during and after EMT6 rechallenge. Whereas all mice were protected from rechallenge in the absence of depletion, this effect was lost in 4/5 CD8-depleted mice, suggesting that protective memory is mediated by CD8^+^ T cells (Supplementary Fig. 2). To determine if this memory was tumor-specific, mice cured from EMT6 tumors were rechallenged simultaneously with EMT6 and TS/A breast cancer cells in opposite fat pads. All cured mice were protected from EMT6 but not from TS/A rechallenge, suggesting that the protective memory is tumor antigen-specific (Fig. 1h). Together these data suggest that combination therapy generates tumor-specific, CD8-dependent protective memory.

### Combination therapy with Entinostat results in increased NHS-IL12 localization in the TME and a sustained peripheral immune stimulatory environment

**Fig. 2:**
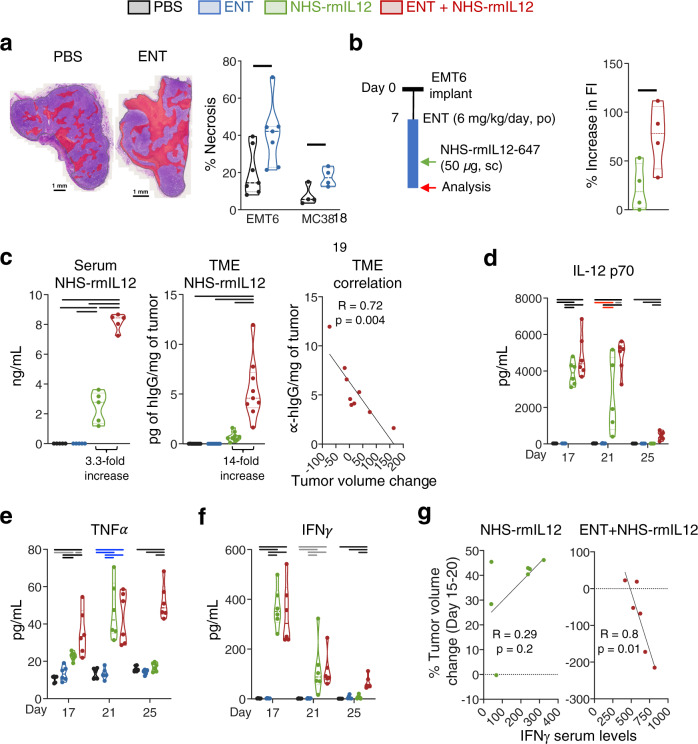
Combination therapy increases NHS-rmIL12 localization in the tumor microenvironment (TME) while promoting a sustained systemic immune stimulatory environment. **a** Representative hematoxylin and eosin (H&E) images of EMT6 tumors treated with PBS or Entinostat (ENT) with areas of necrosis indicated in red. Necrosis quantification in EMT6 (*n* = 7) and MC38 (*n* = 4) tumors after 14 days on ENT. EMT6: *P* = 0.0253; MC38: *P* = 0.0309. **b** EMT6 tumor-bearing mice (*n* = 4) were treated as indicated in the schematic, and % increase in fluorescence (Fl) intensity over the average autofluorescence of PBS- and ENT-treated mice was quantified. *P* = 0.0422. **c** Graphs show quantification of human IgG in the sera (*n* = 5, *P* < 0.0001) or TME supernatant (*n* = 9, *P* < 0.0001) from individual EMT6 tumor-bearing mice on day 21 treated as indicated in Fig. 1a. Correlation of human IgG TME supernatant levels with tumor volume change between days 14 and 20, *n* = 9. Serum levels (*n* = 6) of (**d**) IL-12p70 (*P* < 0.0001), (**e**) TNFα (*P* < 0.0001) and (**f**) IFNγ (d17 and d25, *P* < 0.0001; d21, *P* = 0.0025) at days 17, 21 and 25 post-tumor implant from EMT6 tumor-bearing mice treated as in Fig. 1a. **g** Correlation between change in tumor volume from day 15 to day 20 with IFNγ serum levels on day 21, NHS-rmIL12, *n* = 7; ENT+NHS-rmIL12, *n* = 6. Correlation plots show values from individual mice. Truncated violin plots show values from individual mice with contours denoting kernel density distributions; dashed line, median; and dotted line, interquartile range. Data in panels (**a**, **b**) and day 17 cytokine levels are from one experiment. All remaining data are representative of pooled data from two experiments conducted independently with similar results. Unpaired Student’s *t* test (**a**, **b**), Pearson’s correlation coefficient (**c** right panel, **g**), ordinary one-way ANOVA with Tukey’s multiple comparisons test (**c**–**f**). Statistics are all two-sided. Grey = *p* < 0.05, red = *p* < 0.01, blue = *p* < 0.001, black = *p* < 0.0001. Scale bar: 1 mm. Source data are provided as a Source Data file.

We postulated that superior antitumor efficacy of combination therapy is driven by Entinostat-inducing necrosis and NHS-rmIL12 localizing to the TME by binding exposed DNA in necrotic areas^25^. Quantification of necrosis in EMT6 and MC38 tumors revealed a significant increase after Entinostat treatment (Fig. 2a). This was associated with increased localization of fluorescently labeled NHS-rmIL12 in EMT6 tumors treated with combination therapy (Fig. 2b). Whereas combination therapy significantly elevated (3.3-fold) serum levels of NHS-rmIL12, it promoted heightened (14-fold) localization of NHS-rmIL12 to the TME relative to NHS-rmIL12 alone; this in turn correlated with observed antitumor efficacy in EMT6 tumor-bearing mice (Fig. 2c).

Clinical activity of rhIL-12 has been correlated with the ability to maintain the induction of stimulatory cytokines including IFNγ^12^. Thus, we investigated the impact of NHS-rmIL12 combination therapy on serum cytokines implicated in tumor immune surveillance over time as an indication of the peripheral immune environment and potential clinical biomarkers^12,44^. In the EMT6 model, levels of IL-12p70, IFNγ, TNFα, and IL-10 were examined 2 days after the first (day 17) NHS-rmIL12 dose, as well as 2 (day 21) and 6 days (day 25) after the last NHS-rmIL12 dose. IL-12p70 levels included both endogenous IL-12 and administered NHS-rmIL12. Combination therapy promoted significant levels of serum IL-12p70 relative to either monotherapy or PBS-treated controls at all time points examined (Fig. 2d). Notably, this combination-driven significant elevation of IL-12p70 was still observed 6 days after the last NHS-rmIL12 dosing (day 25), but absent with NHS-rmIL12 monotherapy (Fig. 2d). Similarly, NHS-rmIL12 alone or in combination significantly elevated TNFα (Fig. 2e), IFNγ (Fig. 2f), and IL-10 (Supplementary Fig. 3) on days 17 and 21, but only combination therapy sustained elevated cytokine levels on day 25. Correlative analysis indicated that day 21 IFNγ serum levels were associated with tumor growth inhibition elicited by combination therapy but not by NHS-rmIL12 monotherapy (Fig. 2g). Similar results were observed in MC38-tumor-bearing mice examined on day 21 (Supplementary Fig. 3). In accordance with the inferior antitumor efficacy, rmIL12 combination with Entinostat induced significantly lower levels of serum cytokines (Supplementary Fig. 3). Together, these data suggest that the increased tumor localization of NHS-rmIL12 promoted by Entinostat and a sustained immune stimulatory environment may be key elements of antitumor efficacy and tumor clearance.

### Combination therapy decreases Treg infiltration and promotes tumor engraftment of CD8^+^ T cells

**Fig. 3:**
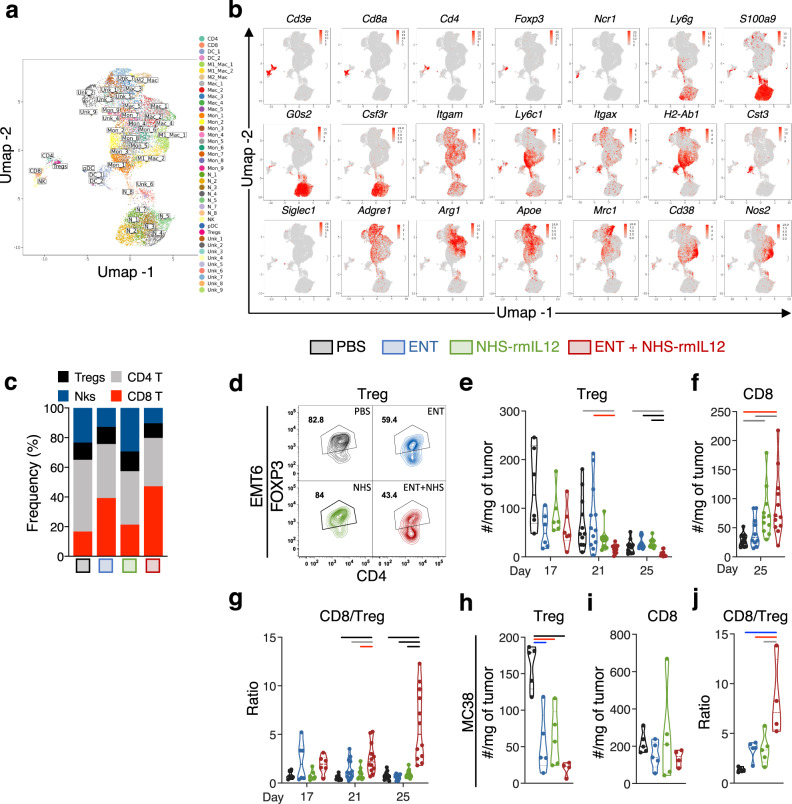
Combination therapy increases CD8^+^ tumor-infiltrating lymphocytes (TILs) while decreasing regulatory T cells (Tregs). EMT6 tumor-bearing mice were treated with PBS, Entinostat (ENT), NHS-rmIL12, or ENT+NHS-rmIL12 (combo) as depicted in Fig. 1a. Two days after the last NHS-rmIL12 dose (day 21), tumor-infiltrating CD45^+^ cells were profiled by scRNAseq (*n* = 5 tumors/group) and flow cytometry. Shown are merged UMAP plots of (**a**) identified immune subsets, (**b**) selected marker gene expression, and (**c**) frequencies of lymphoid subsets from scRNAseq analysis. Each dot represents one single cell and is colored according to cell clusters (**a**) or in red (**b**). **d** Representative flow cytometric analysis of FoxP3 expression on CD4^+^ T cells in EMT6 tumors. Flow cytometry quantification of (**e**) Tregs (d17: *n* = 6; d21: PBS and NHS-rmIL12, *n* = 11; ENT and combo, *n* = 12, *P* = 0.0055; d25: *n* = 11, except combo, *n* = 12, *P* < 0.0001), (**f**) CD8^+^ T cells (PBS and combo, *n* = 12; ENT, *n* = 10; NHS-rmIL12, *n* = 11; *P* = 0.0013) and (**g**) ratio of CD8^+^ T cells to Tregs in EMT6 tumors on days 17 (*n* = 6), 21 (PBS and NHS-rmIL12, *n* = 11; ENT and combo, *n* = 12; *P* < 0.0001), and 25 (*n* = 12 except ENT, *n* = 10; *P* < 0.0001) post-tumor implant. **h**–**j** MC38-tumor-bearing mice were treated as indicated in Supplementary Fig. 4b. Quantification of (**h**) number of Tregs (*P* < 0.0001), (**i**) CD8^+^ T cells and (**j**) ratio of CD8^+^ T cells to Tregs (*P* = 0.0009) in MC38 tumors (*n* = 5, except combo, *n* = 4) at day 21 post-tumor implant by flow cytometry. Truncated violin plots show values from individual mice with contours denoting kernel density distributions; dashed line, median; and dotted line, interquartile range. One-way ANOVA with Tukey’s multiple comparisons test. Statistics are all two-sided. Grey = *p* < 0.05, red = *p* < 0.01, blue = *p* < 0.001, black = *p* < 0.0001. The data from scRNA-seq, EMT6 flow cytometry on day 17, and MC38 flow cytometry are from single independent experiments. All day 21 and remaining day 25 data are representative of pooled data from two experiments conducted independently with similar results. Source data are provided as a Source Data file.

To better understand the mechanism associated with tumor clearance elicited by NHS-rmIL12 combination therapy, we performed a comprehensive and unbiased analysis of the EMT6 tumor immune transcriptome at the single-cell level. Two days after the last NHS-rmIL12 dose (day 21), we performed scRNAseq analysis on CD45^+^ cells isolated from tumors treated with PBS (*n* = 1314 cells sequenced), Entinostat (*n* = 2463 cells sequenced), NHS-rmIL12 (*n* = 1401 cells sequenced) and combination therapy (*n* = 4743 cells sequenced)^45^. All cells (*n* = 9921) were clustered into unbiased cell-type classifications using the Seurat single-cell analysis R package. Dimension reduction analysis identified 40 distinct immune clusters, visualized with Uniform Manifold Approximation and Projection (UMAP; Fig. 3a), and classified as the following populations: one cluster of CD8 T cells expressing *Cd3e* and *Cd8a*, one cluster of non-regulatory CD4 T cells expressing *Cd3e* and *Cd4* and devoid of the transcription factor forkhead box P3 (*Foxp3*), one cluster of CD4 Tregs expressing *Foxp3*, one cluster of NK cells expressing natural cytotoxicity triggering receptor (*Ncr1*), eight clusters (N_1 to N_8) of polymorphonuclear cells/neutrophils expressing lymphocyte antigen 6 complex locus G6D (*Ly6g*), S100 calcium-binding A9 (*S100a9*), G0/G1 switch 2 (*G0s2*) and colony stimulating factor 3 receptor (*Csf3r*). Nine monocyte clusters (Mon_1 to Mon_9) were identified expressing integrin alpha M (*Itgam*) and lymphocyte antigen 6 complex locus C1 (*Ly6c*), 2 clusters of conventional dendritic cells (DC_1 and DC_2) expressing integrin alpha X (*Itgax*), histocompatibility 2 class II antigen A beta 1 *(H2ab1)*, and cystatin C (*Cst3*), and 1 minor cluster of plasmacytoid dendritic cells (pDC) expressing sialic acid binding Ig-like lectin H (*Siglech*). Eight macrophage clusters expressing *Itgam*, adhesion G protein-coupled receptor E1 (*Adgre1*), arginase 1 (*Arg1*), apolipoprotein E (*Apoe*), mannose receptor C-type 1 (*Mrc1*), cluster of differentiation 38 (*Cd38*), and nitric oxide synthase 2 (*Nos2*) were identified. Nine myeloid clusters expressing a range of macrophage- and monocyte-associated genes such as *Itgam*, *Cd68*, *Csf1r*, *Cxcl9*, and *Cx3cr1* were not classified (Unk_1 to Unk_9) (Fig. 3b, Supplementary Tables 1, 2, Supplementary Fig. 3).

Transcriptome analysis of all lymphoid clusters indicated CD8 T cells as the main lymphocyte subset infiltrating the tumor upon combination therapy, with concomitant reduction of NK, CD4, and Treg clusters relative to PBS-treated tumors (Fig. 3c). To probe these findings at the protein single-cell level, we performed a comprehensive flow cytometry-based examination of tumor immunome over time. Combination therapy promoted a significant and sustained reduction of CD4^+^FoxP_3_^+^ Tregs in the EMT6 TME (Fig. 3d, e), without significantly affecting FoxP3^−^ CD4^+^ tumor-infiltrating lymphocytes (TILs) (Supplementary Fig. 4). However, combination therapy promoted a significant increase in CD8^+^ TILs during tumor resolution (day 25) (Fig. 3f and Supplementary Fig. 4), with a gradual and significant increase in CD8^+^ TIL to Treg ratio (Fig. 3g). Similar results were observed in MC38 tumors (Fig. 3h–j and Supplementary Fig. 4), indicating a significant shift towards a more inflamed, less suppressive TME.

### Combination therapy elicits sustained activation and function of CD8^+^ T cells in the periphery and the TME

**Fig. 4:**
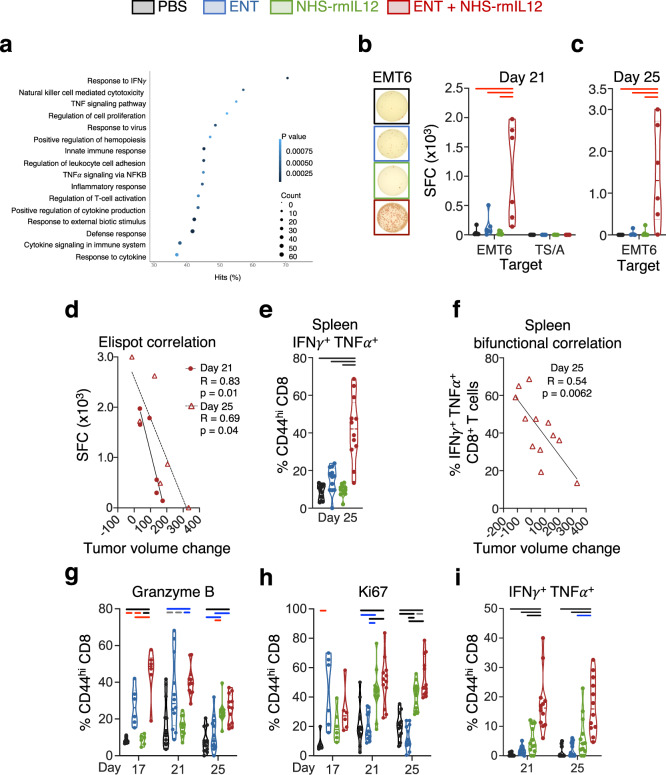
Combination therapy promotes a sustained increase in CD8^+^ tumor-infiltrating lymphocyte (TIL) activation and function. EMT6 tumor-bearing mice were treated with PBS, Entinostat (ENT), NHS-rmIL12 or ENT+rmNHS-IL12 as depicted in Fig. 1a. **a** Selected list of gene pathways upregulated on CD8^+^ TILs from combination-treated mice relative to PBS controls by scRNASeq. **b** Representative image of ELISPOT wells of day 21 splenocytes from each treatment group cultured with EMT6 tumor cells. Graphs show IFNγ spot forming cells (SFC) from splenocytes of individual mice (*n* = 6) on day 21 (EMT6, *P* = 0.0008; TS/A, *P* = 0.7239) or (**c**) day 25 (*P* = 0.0008) post-tumor implant incubated with EMT6 or TS/A tumor cells. Data expressed per 5 × 10^5^ splenocytes/well. **d** Correlation between day 21 and 25 ELISPOT data and tumor volume change between days 14 to 18 and days 21 to 24 for each combination-treated mouse, respectively. **e** Graph shows IFNγ/TNFα double producing, bifunctional CD8^+^ splenic T cells on day 25 post-tumor implant (*n* = 12, *P* < 0.0001). **f** Correlation of cytokine data in panel (**e**) to tumor volume change between days 14 to 18 for each combination-treated mouse. Expression of (**g**) granzyme B (*P* < 0.0001) and (**h**) Ki67 (d17, *P* = 0.0054; d21 and d25, *P* < 0.0001) in CD8^+^ TILs as a measure of T-cell activation on days 17 (*n* = 6), 21 (*n* = 12), and 25 (*n* = 12) post-tumor implant. **i** Frequency of IFNɣ and TNFα double producing, bifunctional CD8^+^ TILs without ex vivo stimulation on days 21 (*P* < 0.0001) and 25 (*P* < 0.0001) post-tumor implant, *n* = 12. Correlation plots show values from individual mice. Truncated violin plots show values from individual mice with contours denoting kernel density distributions; dashed line, median; and dotted line, interquartile range. For correlations, Pearson’s correlation coefficient was performed. One-way ANOVA with Tukey’s multiple comparisons test. Statistics are all two-sided. Grey = *p* < 0.05, red = *p* < 0.01, blue = *p* < 0.001, black = *p* < 0.0001. scRNA-seq, day 25 ELISPOT and day 17 data are from single independent experiments. Day 21 and remaining day 25 data are representative of pooled data from two experiments conducted independently with similar results. Source data are provided as a Source Data file.

Next, we investigated the functional status of infiltrated and peripheric lymphocytes. Transcriptome analysis of EMT6 CD8 TILs by scRNAseq revealed that combination therapy promoted significant activation of multiple pathways related to T-cell function, including defense response, response to IFNγ, cytokine signaling and response, positive regulation of cytokine production, TNF signaling pathway, and T-cell proliferation (Fig. 4a and Supplementary Data File 1). In the periphery, combination therapy elicited a significant EMT6 tumor-specific IFNγ response (Fig. 4b, day 21), sustained 6 days after treatment cessation (Fig. 4c, day 25); both correlated with tumor size reduction (Fig. 4d). This sustained immune activation elicited by combination therapy was further supported by functional analysis demonstrating a significant increase in activated bifunctional CD8^+^ T cells producing both IFNγ and TNFα at day 25 (Fig. 4e), which also correlated with tumor size reduction (Fig. 4f). To ascertain if this pattern of sustained T-cell activation was present in the TME, we examined TIL phenotype and function throughout the course of treatment (days 17 and 21) and 2 days post-treatment cessation (day 25). Examination of EMT6 CD8^+^ TILs revealed that combination therapy promoted a significant and sustained increase in granzyme B (Fig. 4g) and Ki67 (Fig. 4h), which was also observed in CD4^+^ TILs at day 25 (Supplementary Fig. 4), suggesting increased cytotoxicity and proliferative capacity, respectively. Notably, these effects were not observed in MC38 tumors that displayed reduced infiltration of effector FoxP_3_^−^CD4^+^ TILs (Supplementary Fig. 4). Functional analysis of activated CD8^+^ TILs demonstrated a significant and sustained presence of bifunctional CD8^+^ lymphocytes producing both IFNγ and TNFα in the absence (Fig. 4i) and presence (Supplementary Fig. 4) of ex vivo T-cell receptor stimulation. Collectively, this demonstrated that combination therapy elicited a sustained reprogramming of the immunome in both the periphery and the tumor, resulting in a functionally inflamed TME correlating with tumor resolution.

### Combination therapy educates innate and adaptive immunity to favor tumor engraftment of CD8^+^ TILs

**Fig. 5:**
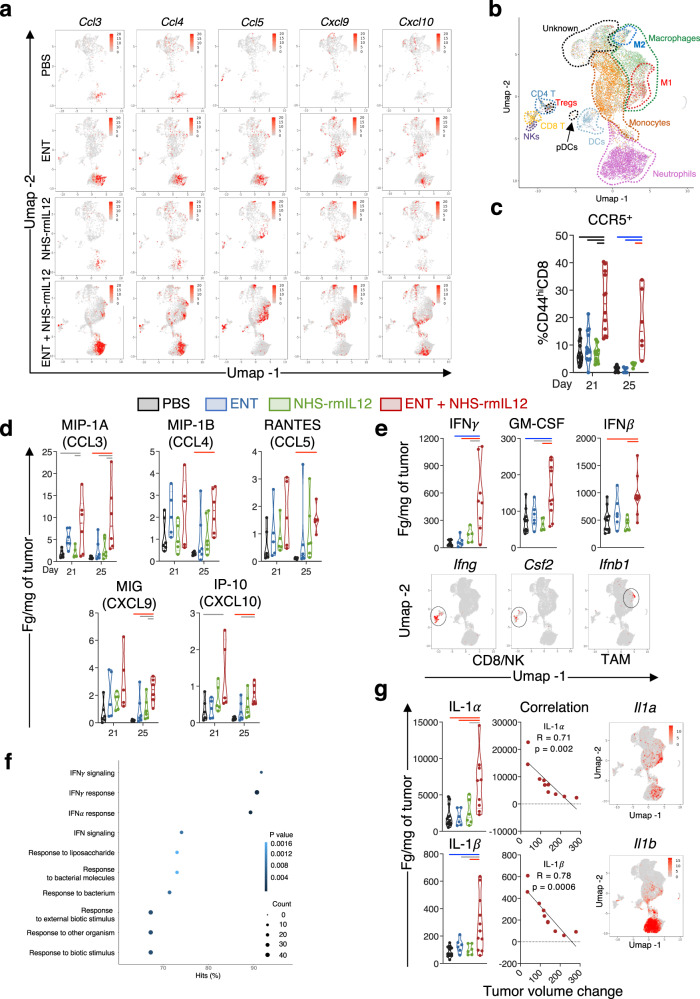
Combination therapy increases tumor chemokines associated with T-cell infiltration. EMT6-tumor-bearing mice were treated with PBS, Entinostat (ENT), NHS-rmIL12 or ENT+NHS-rmIL12 (combo) as depicted in Fig. 1a. Two days after the last NHS-rmIL12 dose (day 21), tumor-infiltrating CD45^+^ cells were analyzed by scRNAseq and flow cytometry. UMAP plots show (**a**) expression of designated chemokine genes associated with T-cell chemotaxis, and (**b**) major immune subset clusters identified by scRNAseq. Each dot represents one single cell and is colored in red (**a**) or according to major immune cell subsets identified (**b**). **c** CCR5 expression on splenic CD44^hi^CD8^+^ T cells by flow cytometry on days 21 (*n* = 12, *P* < 0.0001) and 25 (*n* = 6, *P* = 0.0001) post-tumor implant. Proteomic quantification of (**d**) CCR5- and CXCR3-associated chemokines (*n* = 6; d25: CCL3, *P* = 0.0066; CCL4, *P* = 0.0111; CCL5, *P* = 0.0047; CXCL9, *P* = 0.0019; CXCL10, *P* = 0.0051), and (**e**) designated cytokines (IFNγ: PBS, *n* = 10; ENT, *n* = 6; NHS-rmIL12, *n* = 5; combo, *n* = 8; *P* = 0.0019; GM-CSF: *n* = 6, except PBS and combo, *n* = 12; *P* = 0.0005; IFNβ: *n* = 6, except PBS and combo, *n* = 12; P = 0.0008) in tumor microenvironment (TME) supernatant on designated days post-tumor implant. Lower panels show UMAP gene expression clusters for the respective encoding genes. **f** Upregulated GO/HALLMARK gene pathways elicited by combination therapy in combined neutrophil gene clusters relative to PBS controls. **g** Proteomic quantification of designated cytokines (left panels, IL1α: PBS, *n* = 12; ENT and NHS-rmIL12, *n* = 6; combo, *n* = 9; *P* = 0.0016; IL1β: PBS, *n* = 12; ENT and NHS-rmIL12, *n* = 6; combo, *n* = 11; *P* = 0.0015), correlation between cytokine levels and tumor volume change between days 14 and 18 for each combination-treated mouse (middle panels), and UMAP gene expression of designated encoding genes (right panels). Correlation plots show values from individual mice. Truncated violin plots show values from individual mice with contours denoting kernel density distributions; dashed line, median; and dotted line, interquartile range. Pearson’s correlation coefficient (**g**, middle panels), and ordinary one-way ANOVA with Tukey’s multiple comparisons test (**c**–**g**). Grey = *p* < 0.05, red = *p* < 0.01, blue = *p* < 0.001, black = *p* < 0.0001. Statistics are all two-sided. The data from RNA-seq, and TME chemokine proteins on day 25 are from single independent experiments. All day 21 and remaining day 25 data are representative of pooled from two experiments conducted independently with similar results. Source data are provided as a Source Data file.

To get a better insight into the mechanism driving CD8 tumor infiltration, we interrogated the scRNAseq gene expression and protein levels of chemokines associated with T-cell migration and chemotaxis^7^. Combination therapy significantly upregulated multiple genes encoding T-cell attracting chemokines, including *Ccl3, Ccl4, Ccl5, Cxcl9*, and *Cxcl10* (Fig. 5a). Gene mapping analysis indicated that this upregulation was mostly contributed by neutrophil (*Ccl3, Ccl4, Cxcl10*) and macrophage (*Ccl4, Ccl5, Cxcl9, Cxcl10*) clusters, with the contribution of CD8 T cell (*Ccl3, Ccl4, Ccl5*) and monocyte (*Ccl4, Cxcl9, Cxcl10*) clusters (Fig. 5b). This was associated with a gradual and significant increase in CD44^hi^ CCR5-expressing splenic CD8^+^ T cells (Fig. 5c and Supplementary Fig. 4) and an elevation in the cognate ligand proteins CCL3, CCL4, and CCL5, as well as the CXCR3 ligands CXCL9 and CXCL10 in the TME (Fig. 5d). Collectively these data suggested that combination therapy modulated the myeloid compartment resulting in tumor engraftment of CD8^+^ TILs. Analysis of other myeloid subsets did not reveal significant or consistent modulation of tumor infiltration of dendritic cells, including CD103^+^ DCs, neutrophils or monocytic MDSCs (Supplementary Fig. 4).

To further dissect this cross-talk between tumor lymphoid and myeloid subsets, we probed the TME for cytokines associated with myeloid activation^46^. We observed a significant elevation of IFNγ, GM-CSF, and IFNβ proteins in the TME upon combination therapy, mapped to CD8 and NK (*Ifng, csf2*), and macrophage (*Ifnb*) gene clusters identified by scRNAseq (Fig. 5e). IFNγ, GM-CSF, and IFNβ have shown to induce an interferon-stimulated gene signature in tumor-associated neutrophils (TANs), reprogramming TANs to an antitumor program^46^. Analysis of all TAN gene clusters combined revealed that upon combination therapy, the top 10 significantly upregulated pathways were associated with IFNγ signaling and response, IFNα response, response to lipopolysaccharide and bacterium (Fig. 5f and Supplementary Data File 2). This indicated that combination therapy reprogrammed (TANs) towards a proinflammatory interferon-responsive gene program, hallmark of antitumor neutrophils^46^. To probe if neutrophils contributed to antitumor activity elicited by combination therapy, we quantified protein levels of IL1α and IL1β in the TME. Whereas IL1α is known to promote leukocyte recruitment, IL1β has been shown to support the survival and effector function of neutrophils and macrophages^46^. Upon combination therapy, we observed a significant increase in IL1α and IL1β protein levels in the TME, both correlating with tumor regression (Fig. 5g). In addition, *Il1a* and *Il1b* gene expression mapped to neutrophil clusters by scRNAseq, collectively suggesting that neutrophils are actively contributing to antitumor effects.

Furthermore, gene ontology analysis indicated significant activation of multiple pathways pertaining to IFNα response, IFNα/β and IFNγ signaling and response, and cytokine signaling and response in monocyte gene clusters (Supplementary Fig. 5 and Supplementary Data File 3). Collectively, these findings suggest that combination therapy promotes a proinflammatory tumor immune environment through a cooperative lymphoid/myeloid loop encompassing reprogramming of multiple myeloid populations, conducive to tumor resolution.

### Combination therapy reprograms myeloid cell differentiation in the TME, resulting in a significant shift in M1/M2 macrophage balance favoring tumor resolution

**Fig. 6:**
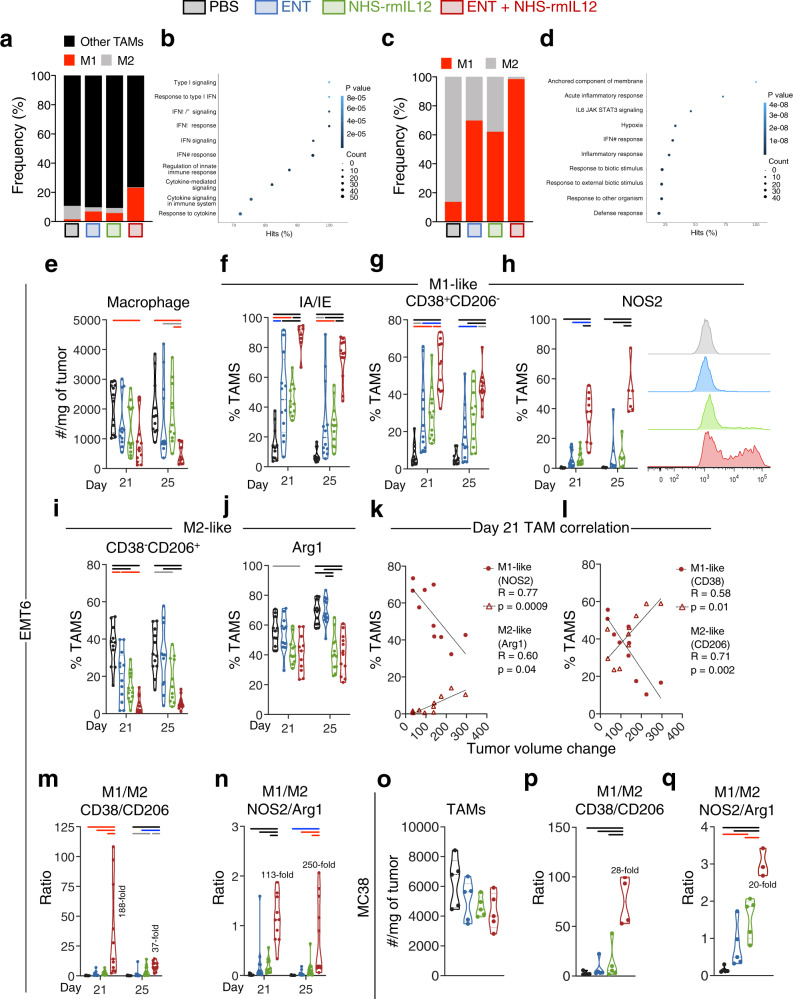
Combination therapy promotes M1-like over M2-like tumor-associated macrophages (TAMs). EMT6 tumor-bearing mice were treated as depicted in Fig. 1a. **a** Frequencies of TAM populations identified by scRNAseq. **b** Top 10 upregulated gene pathways on TAM gene clusters identified by scRNAseq as not M1- or M2-like from combination-treated mice relative to PBS controls. **c** Relative frequency of M1- and M2-like TAMs by scRNAseq. **d** Top 10 upregulated gene pathways on M1-like TAM gene clusters identified by scRNAseq from combination-treated mice relative to PBS controls. **e** Flow cytometry quantification of TAM infiltration on days 21 (*n* = 12, except ENT, *n* = 11; *P* = 0.0056) and 25 (*n* = 12, except ENT and NHS-rmIL12, *n* = 11; *P* = 0.0015) post-tumor implant. Expression of M1-like markers (**f**) IA/IE (d21: PBS and NHS-rmIL12, *n* = 12; ENT, *n* = 11; combo, *n* = 8; *P* < 0.0001; d25: *n* = 12, except ENT, *n* = 11; *P* < 0.0001), (**g**) CD38^+^ CD206^-^ (d21: *n* = 12, except PBS and combo, *n* = 11; *P* < 0.0001; d25: *n* = 12, except PBS, *n* = 11; *P* < 0.0001) and (**h**) NOS2 (d21: *n* = 10, except NHS-rmIL12, *n* = 11; *P* < 0.0001; d25: *n* = 6, *P* < 0.0001) on TAMs. Representative histograms of NOS2 flow cytometry data from day 21 post-tumor implant. Expression of M2-like markers (**i**) CD38^-^CD206^+^ (d21: *n* = 12, except PBS and combo, *n* = 11; *P* < 0.0001; d25: *n* = 12, except NHS-rmIL12, *n* = 11; *P* < 0.0001) and (**j**) Arg1 (d21: *n* = 11, except combo, *n* = 10; *P* = 0.0085; d25: *n* = 12, *P* < 0.0001) on TAMs. Correlation of M1-like or M2-like TAM frequency to tumor volume change between days 14 to 18 for each combination-treated mouse using the markers NOS2/Arg1 (**k**) or CD38/CD206 (**l**). M1 to M2 ratios using the markers CD38/CD206 (d21: PBS and combo, *n* = 10; ENT, *n* = 9; NHS-rmIL12, *n* = 11; *P* = 0.0004; d25: PBS and combo, *n* = 11; ENT, *n* = 10; NHS-rmIL12, *n* = 12; *P* < 0.0001) (**m**) or NOS2/Arg1 (d21: *n* = 11, except combo, *n* = 10; *P* < 0.0001; d25: PBS and combo, *n* = 12; ENT and NHS-rmIL12, *n* = 11; *P* = 0.0002) (**n**). TAM infiltration (*n* = 5) (**o**) and M1/M2 ratios on day 21 post-tumor implant using the markers CD38/CD206 (*n* = 5, except combo, *n* = 4; *P* < 0.0001) (**p**) or NOS2/Arg1 (*n* = 5, except combo, *n* = 3; *P* < 0.0001) (**q**) in MC38 tumors treated as indicated in Supplementary Fig. 4b. Correlation plots show values from individual mice. Truncated violin plots show values from individual mice with contours denoting kernel density distributions; dashed line, median; and dotted line, interquartile range. Pearson’s correlation coefficient (**l**), one-way ANOVA with Tukey’s multiple comparisons test (**e**–**j**, **m**–**q**). Statistics are all two-sided. Grey = *p* < 0.05, red = *p* < 0.01, blue = *p* < 0.001, black = *p* < 0.0001. scRNA-seq and MC38 data are from single independent experiments. EMT6 day 21 and 25 data representative of pooled from two experiments conducted independently with similar results. Combo, ENT+NHS-rmIL12. Source data are provided as a Source Data file.

To get a deeper insight into the myeloid reprograming elicited by combination therapy, we probed the scRNAseq transcriptome from EMT6 tumors for alterations in tumor-associated macrophages (TAMs). We identified eight TAM clusters, including two proinflammatory M1-like clusters expressing *Cd38*, *Nos2*, *Cd40*, and *Cd86*, one regulatory M2-like cluster expressing *Mrc1* and *Cd163*, and five undefined TAM clusters with gradient expression of multiple other macrophage-associated genes, including *Adgre1*, *Itgam*, *Apoe*, *Arg1*, *Mmp12*, *Csf1r*, *Cd68*, *Lgals3*, and *Cxcl10* (Supplementary Figs. 3 and 5 and Supplementary Tables 1 and 2). Frequency analysis indicated that the majority of identified TAMs were not M1- or M2-like, in agreement with known plasticity of macrophages in the TME (Fig. 6a). Analysis of the top 10 GO/KEGG/REACTOME/HALLMARK pathways modulated in these combined intermediate clusters (Mac_1 to Mac_5) in response to combination therapy indicated significant upregulation of proinflammatory pathways, including interferon signaling, response to IFNα, β, and γ, cytokine signaling and response to cytokine, and regulation of innate immune response (Fig. 6b and Supplementary Data File 4). Analysis of M1 and M2-like clusters suggested a significant increase in the proinflammatory M1 to the regulatory M2 balance in the TME upon combination therapy, supportive of tumor resolution (Fig. 6c and Supplementary Fig. 5). Pathway analysis of M1-like gene clusters indicated that combination therapy significantly upregulated multiple pathways supportive of an antitumor program^47,48^, including interferon gamma response, inflammatory response, and IL6/JAK/STAT3 signaling (Fig. 6d and Supplementary Data File 5).

This finding prompted us to examine the effects of combination therapy on TAMs and its impact on tumor resolution. Microenvironment signals, such as IL-12 and IFN-γ, can skew polarization of M2 pro-tumor TAMs to M1-like, with tumor-suppressive properties^16^. We observed that combination therapy significantly decreased the overall TAM infiltration in EMT6 tumors (Fig. 6e and Supplementary Fig. 6). However, combination therapy elicited a significant and sustained increase in antitumor M1-like TAMs, as demonstrated by augmented IA/IE (Fig. 6f), CD38 (Fig. 6g), NOS2 (Fig. 6h), as well as CD80 and CD86 (Supplementary Fig. 6) expression, initially observed 2 days after initiation of NHS-IL12 dosing (Supplementary Fig. 6). This was paralleled with a significant reduction of pro-tumor M2-like CD206^+^ (Fig. 6i and Supplementary Fig. 6) and Arg1^+^ (Fig. 6j and Supplementary Fig. 6) TAMs. Further analysis indicated IL1α, IL1β, and GM-CSF levels in the TME to be positively correlated with M1 infiltration (Supplementary Fig. 6). In addition, M1 TAM infiltration was positively correlated with tumor reduction, with the oppositive effect observed with M2 TAMs (Fig. 6k, l).

Overall, combination therapy dramatically increased the M1/M2 ratio in the EMT6 TME during and after therapy cessation (Fig. 6m, n and Supplementary Fig. 6), with similar results observed in MC38 TAMs (Fig. 6o–q and Supplementary Fig. 6). This shift in the TAM balance favoring tumor resolution elicited by combination therapy was further supported by elevated expression of the “eat-me signal” calreticulin over the “don’t-eat me signal” CD47 on CD45^neg^ non-immune/tumor cells in the TME upon combination therapy (Supplementary Fig. 6), collectively suggesting a pattern conducive to macrophage-mediated tumor clearance^49^. Collectively, these data suggest that tumor eradication elicited by the combination therapy is associated with a dynamic reprogramming in the TME myeloid landscape with significant M1-like polarization, correlating to antitumor efficacy.

### Innate and adaptive cross-talk determines tumor eradication

**Fig. 7:**
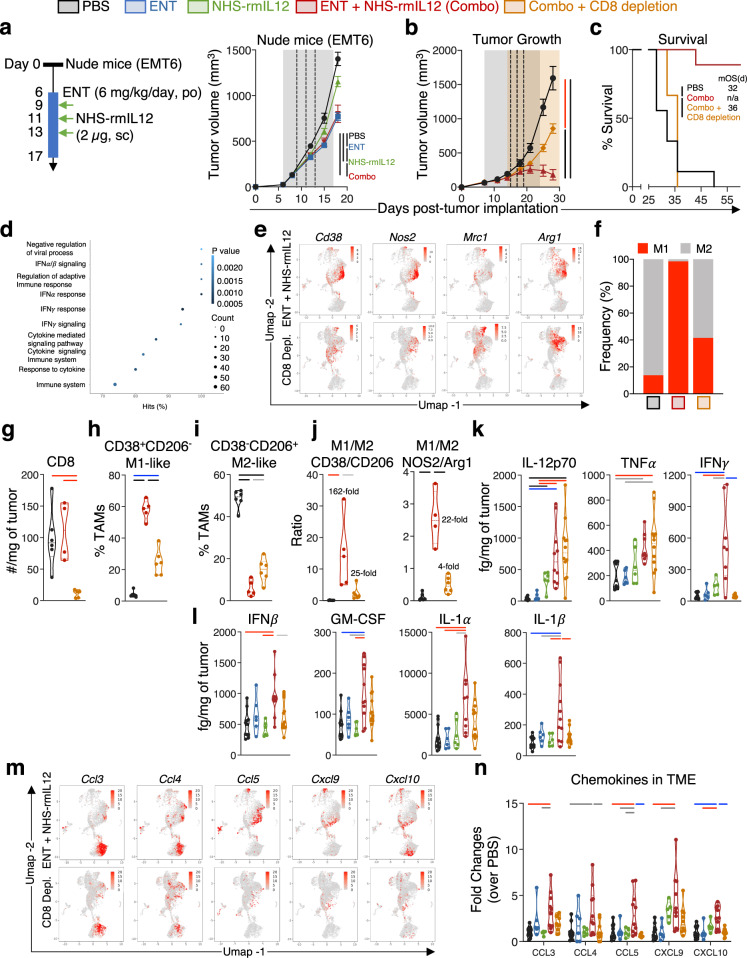
Tumor eradication requires CD8^+^ TILs to efficiently polarize M1-like tumor-associated macrophages (TAMs). **a** Tumor growth of EMT6 tumor-bearing athymic nu/nu mice (PBS and ENT, *n* = 10; NHS-rmIL12, *n* = 8; combo, *n* = 9; *P* < 0.0001) treated with Entinostat (ENT) and/or NHS-rmIL12 as indicated in the schematic. **b** Tumor growth (*P* < 0.0001) and (**c**) survival (*P* < 0.0001) in syngeneic Balb/c mice after treatment with ENT+NHS-rmIL12 as in Fig. 1a with or without CD8 depletion, *n* = 9. Inset indicates median overall survival (mOS). For all tumor growth curves, gray shaded area indicates Entinostat treatment and dashed lines are NHS-rmIL12 doses. CD8 depletion period is indicated with orange shading. **d** Top 10 pathways downregulated in TAM gene clusters from CD8-depleted mice treated with combination therapy vs. undepleted. **e** Expression by scRNAseq of select M1 (*Cd38, Nos2*) and M2-associated (*Mrc1, Arg1*) genes in CD45^+^ cells isolated from CD8-depleted or undepleted EMT6 tumors. Each red dot represents one single cell. **f** Effect of CD8 depletion in the relative frequency of M1- and M2-like TAMs by scRNAseq. Mice from depletion study were analyzed 21 days after tumor implant and (**g**) CD8 tumor-infiltrating lymphocytes (TIL) (PBS, *n* = 6; combo, *n* = 4, combo/CD8dep, *n* = 5; *P* = 0.0028), (**h**) M1-like CD38^+^ CD206^-^ TAMs (PBS and combo/CD8dep^,^ *n* = 6^;^ combo, *n* = 5; *P* < 0.0001), (**i**) M2-like CD38^-^CD206^+^ TAMs (PBS and combo/CD8dep, *n* = 6; combo, *n* = 5; *P* < 0.0001), and (**j**) the ratio of M1/M2 TAMs using (i) CD38/CD206 (PBS and combo/CD8dep, *n* = 6; combo, *n* = 5; *P* = 0.0030) or (ii) NOS2/Arg1 (PBS and combo/CD8dep, *n* = 6; combo, *n* = 4; *P* < 0.0001) was quantified by flow cytometry. **k**–**l** Graphs show quantification of (**k**) IL12p70 (PBS and combo, *n* = 10; ENT and NHS-rmIL12, *n* = 6; combo/CD8dep, *n* = 12; *P* < 0.0001), TNFα (PBS, *n* = 11; ENT and NHS-rmIL12, *n* = 6; combo, *n* = 8; combo/CD8dep, *n* = 12; *P* = 0.0011), IFNγ (PBS, *n* = 10; ENT, *n* = 6; NHS-rmIL12, *n* = 5; combo, *n* = 11; combo/CD8dep, *n* = 12; *P* = 0.0002), and (**l**) IFNβ (PBS, *n* = 11; ENT and NHS-rmIL12, *n* = 6; combo, *n* = 10; combo/CD8dep, *n* = 12; *P* = 0.0009), GM-CSF (PBS, *n* = 11; ENT and NHS-rmIL12, *n* = 6; combo, *n* = 11; combo/CD8dep, *n* = 12; *P* = 0.0005), IL1α (PBS, *n* = 12; ENT and NHS-rmIL12, *n* = 6; combo, *n* = 10; combo/CD8dep, *n* = 12; *P* = 0.0009), and IL1β (PBS, *n* = 12; ENT and NHS-rmIL12, *n* = 6; combo, *n* = 11; combo/CD8dep, *n* = 12; *P* = 0.0004) in tumor microenvironment (TME) supernatant on day 21 post-tumor implant in EMT6 tumor-bearing Balb/c mice. **m**, **n** Effect of EMT6 tumor CD8 depletion on designated chemokines’ (**m**) gene expression by RNAseq and (**n**) CCL3 (*P* = 0.0021), CCL4 (*P* = 0.0146), CCL5 (*P* = 0.0007), CXCL9 (*P* = 0.0014), and CXCL10 (*P* = 0.0002) protein levels in TME supernatant (PBS, *n* = 11; ENT, *n* = 6; NHS-rmIL12, *n* = 5; combo and combo/CD8dep, *n* = 10). Each dot represents one single cell (**e**). Correlation plots show values from individual mice. Truncated violin plots show values from individual mice with contours denoting kernel density distributions; dashed line, median; and dotted line, interquartile range. For survival, log-rank (Mantel–Cox) was used for comparisons. One-way ANOVA with Tukey’s multiple comparisons test was used for all comparisons except two-way ANOVA was used for average tumor growth comparison. Statistics are all two-sided. Grey = *p* < 0.05, red = *p* < 0.01, blue = *p* < 0.001, black = *p* < 0.0001. Data from single independent experiments (**b**–**f**, **m**) or pooled from two experiments conducted independently with similar results (**a**, **g**–**l**, **n**). Combo, ENT+NHS-rmIL12. Source data are provided as a Source Data file.

To determine if myeloid reprogramming was determinant to the antitumor effects of combination therapy or whether T cells sufficed, athymic nu/nu mice, which lack CD4^+^ and CD8^+^ T cells, were utilized. In these mice, combination therapy inhibited EMT6 tumor growth only by 46.1%, failing to provide additional tumor control over Entinostat monotherapy (Fig. 7a and Supplementary Fig. 7); this is in contrast with the complete tumor resolution and survival benefit observed in syngeneic mice. In accordance, depletion of CD8^+^ T cells in syngeneic mice treated with combination therapy precluded EMT6 tumor eradication (Fig. 7b and Supplementary Fig. 7) and survival benefit (Fig. 7c). This indicated that other immune effectors were necessary for complete tumor eradication, including a potential role for M1 TAMs. Transcriptome analysis of CD8-depleted mice treated with combination therapy revealed that in the absence of CD8^+^ TILs, TAMs downregulated multiple pathways related to IFNα, β, and γ, cytokine signaling and response, and regulation of adaptive immune response (Fig. 7d and Supplementary Data File 6), with significant expression loss of M1-associated genes *Cd38* and *Nos2*, and increase in M2-associated genes *Mrc1* and *Arg1* (Fig. 7e), reversing the elevated M1-to-M2 gene expression balance elicited by combination therapy in immune intact mice (Fig. 7f). Flow cytometry analysis of CD8-depleted mice treated with combination therapy revealed that in the absence of CD8^+^ TILs (Fig. 7g), the presence of M1-like TAMs (Fig. 7h and Supplementary Fig. 7) was significantly reduced with the opposite effect observed in M2-like TAMs (Fig. 7i and Supplementary Fig. 7). This resulted in a severe 6-fold reduction in the M1-to-M2 ratio relative to non-depleted mice (Fig. 7j). Similarly, in athymic mice, while combination therapy did not affect overall TAM infiltration (Supplementary Fig. 7), the increase in M1-to-M2 balance was significantly less (8.5-fold) (Supplementary Fig. 7) than observed in syngeneic (162-fold) mice. This suggested that tumor eradication involved a cooperative mechanism involving CD8^+^ TILs and M1-like TAMs. This is concordant with the elevated levels of M1-polarizing cytokines IL-12, TNFα, and IFNγ (Fig. 7k) observed in the EMT6 TME of syngeneic mice treated with combination therapy. In vitro exposure of unpolarized (M0) bone-marrow-derived macrophages (BMDM) from control EMT6 tumor-bearing mice to IFNγ and TNFα induced significant M1 but not M2 polarization (Supplementary Fig. 7). In the absence of CD8^+^ TILs, the elevated levels of IFNγ were abrogated despite maintained high IL-12 and TNFα levels in the TME (Fig. 7k).

In addition, CD8 depletion negated the increase in TME IFNβ, GM-CSF, IL-1α, and IL-1β induced by combination therapy (Fig. 7l), with all neutrophils significantly downregulating the IFNγ response gene pathway (Supplementary Fig. 7 and Supplementary Table 5). Furthermore, the absence of CD8^+^ TILs negated the increase in the TME *Ccl3, Ccl4, Ccl5, Cxcl9*, and *Cxcl10* chemokine genes (Fig. 7m) and encoded proteins (Fig. 7n) induced by combination therapy. Collectively, these data demonstrate that combination therapy elicits a dynamic cross-talk between CD8^+^ TILs, macrophages, and neutrophils, resulting in a robust M1 polarization and neutrophil activation, collectively reprogramming the TME into a highly proinflammatory milieu able to clear well-established immunologically cold tumors.

### A biomarker signature elicited by combination therapy correlates to patient CD8^+^ TILs and M1 TAM infiltration and overall survival

**Fig. 8:**
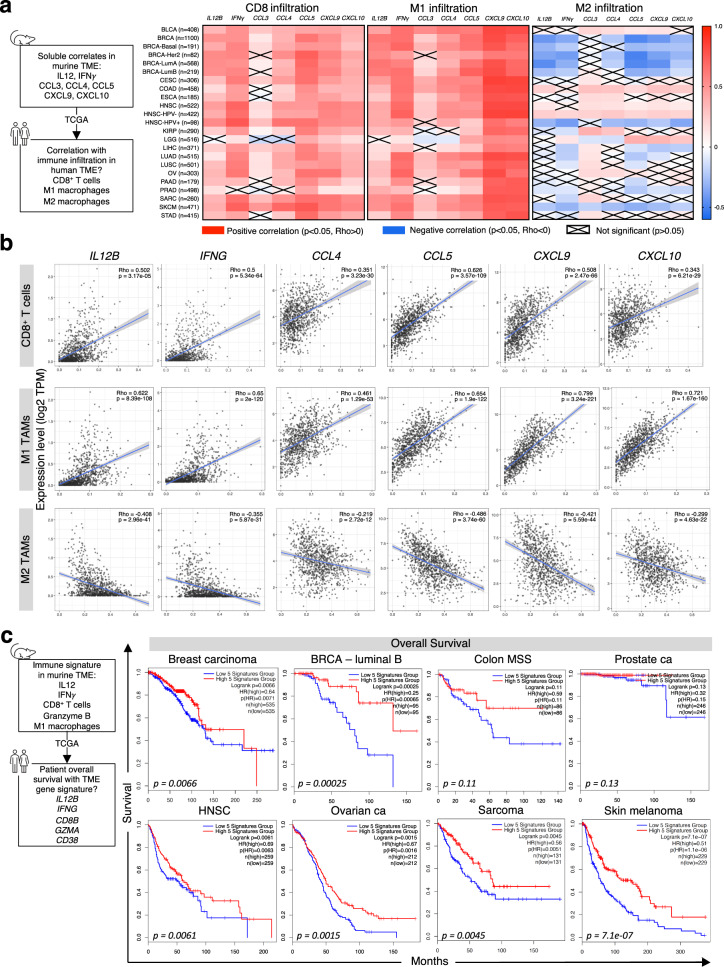
Translational relevance of the murine studies. **a** Heatmap of Spearman’s correlations between genes encoding key immune markers responsible for the antitumor efficacy of NHS-rmIL12 plus Entinostat treatment in murine models, and immune infiltration in various human cancers. Data analysis from designated TCGA datasets was generated using the open-source platform TIMER2.0 and heat maps generated using GraphPad Prism 7. red = Spearman Rho >0 corresponds to positive correlation; blue = Spearman Rho <0 corresponds to negative correlation; not significant Spearman Rho are crossed out. **b** Corresponding dot plot of two-tailed Spearman’s correlation of the TCGA breast cancer data set (BRCA, *n* = 1100). Line represents best-fitting regression line with 95% confidence interval (grey shading). **c** Correlation between the key immune signature (IL12B, IFNG, CD8B, GZMA, CD38) observed in murine models with clinical overall survival of human patients with various cancer types. Kaplan–Meier curves were generated using the open-source platform GEPIA2, with a median high/low 50% cutoff. BLCA, bladder cancer; BRCA, breast cancer; CESC, cervical squamous cell cancer; COAD, colon adenocarcinoma; ESCA, esophageal carcinoma; HNSC, head and neck squamous carcinoma; KIRP, kidney renal papillary cell carcinoma; LGG, low-grade glioma; LIHC, liver hepatocellular carcinoma; LUAD, lung adenocarcinoma; LUSC, lung squamous cell adenocarcinoma; OV, ovarian cancer; PAAD, pancreatic adenocarcinoma; PRAD, prostate adenocarcinoma; SARC, sarcoma; SKCM, skin cutaneous melanoma; STAD, stomach adenocarcinoma. Mouse and human icons (**a**, **c**) were imported from powerpoint (Microsoft Powerpoint for Mac v 16.49, Microsoft).

Our findings identified several soluble protein correlates in the TME associated with increased infiltration of CD8^+^ T cells, high M1/M2 polarization, and neutrophil activation, namely IL-12, IFNγ, CCL3, CCL4, CCL5, CXCL9, and CXCL10. To determine if these mechanistic findings could translate to data from cancer patients, we interrogated multiple Tumor Genome Cancer Atlas (TCGA) datasets to examine if the genes transcribing these cytokines and chemokines were associated with infiltration of CD8^+^ T cells, M1, and M2 macrophages in human tumors (Fig. 8a). We observed significant positive correlations across 23 different tumor types, including breast cancer, between CD8^+^ T-cell infiltration and all probed chemokines and cytokine genes, with *Ccl3* being the least correlated. In the large majority of tumor types, *Il12b*, *Ifng*, *Ccl4*, *Ccl5*, *Cxcl9*, and *Cxcl10* showed a significant positive correlation with infiltration of M1 macrophages, with *Ccl3* displaying a lesser overall correlation. In contrast, all interrogated genes showed insignificant or negative correlation with tumor infiltration of M2 macrophages across the majority of tumor types, albeit some positive correlation was noted, namely with *Ccl3* and *Ccl4* in colon adenocarcinoma (COAD), esophageal cancer (ESCA), and HPV^‒^ head and neck squamous carcinoma (HNSC).

A more detailed analysis of 1100 patients with breast cancer (BRCA) revealed significant positive correlations between *Il12b*, *Ifng*, *Ccl4*, *Ccl5*, *Cxcl9*, and *Cxcl10* gene expression levels and tumor infiltration of CD8^+^ T cells and M1 macrophages (Fig. 8b). In contrast, expression levels of all these genes correlated negatively with tumor infiltration of M2 macrophages. In addition, in colon adenocarcinoma biopsies, all interrogated genes were positively correlated with immune infiltration, including M2 macrophages (Supplementary Fig. 8).

Collectively, these data suggest that the findings may be of potential clinical value. To further probe this possibility, we identified a biomarker signature in the TME reflective of our main mechanistic findings and associated with antitumor efficacy of NHS-rmIL12 and Entinostat in our preclinical studies, composed by IL12, IFNγ, CD8^+^ T cells, granzyme B, and M1 macrophages. To interrogate its clinical translation value using TCGA datasets, we created a surrogate human gene signature composed by *Il12b*, *Ifng*, *Cd8b*, *Gzma*, and *Cd38*. Probing this gene signature across a range of tumor types revealed that patients harboring high gene expression levels of this signature had a significantly higher overall survival relative to those with low gene expression (Fig. 8c). This was observed in patients with breast carcinoma, particularly in the luminal B subtype, as well as HNSC, ovarian cancer, sarcoma, and melanoma, with a trend observed in patients with microsatellite-stable (MSS) colon or prostate carcinomas. Collectively, these data suggest that combination therapy with NHS-IL12 and Entinostat may translate into clinical benefit, thus warranting clinical evaluation.

## Discussion

Immunotherapy targeting immune checkpoints such as programmed death ligand 1(PD-1) and its ligand (PD-L1) provide limited clinical benefit to most carcinoma patients^1^. The efficacy of immune checkpoint blockade inherently relies on pre-existing antitumor immunity, generally absent in poorly inflamed carcinomas such breast, colorectal, and prostate^3,50^.

Here we presented an alternative approach to convert the TME to a functionally immune inflamed hub by eliciting a robust cross-talk between innate and adaptive immunity. The concerted reprogramming of the TME efficiently sustained tumor destruction, eradicating established murine breast and colorectal tumors. In this study, combining the HDAC inhibitor Entinostat with the immunocytokine NHS-rmIL12 achieved significant tumor resolution in three distinct models with a range of immunogenicity and sensitivity to anti-PD-L1 monotherapy, conferring long-lasting protective immunity in all three models. The absence of protective immunity in EMT6 cured mice against another poorly immunogenic breast tumor model (TS/A)^51^ partially responsive to NHS-rmIL12 plus Entinostat therapy (38% tumor growth inhibition) confirmed protective memory to be tumor specific.

Notably, combination therapy induced significant antitumor efficacy against tumors poorly responsive (EMT6)^27,40^ or resistant (CT26)^52,53^ to PD-L1 inhibition, suggesting this combination may be a viable option to treat patients harboring innate or acquired resistance to checkpoint inhibition.

Here we demonstrated that effectively targeting IL12 to the TME is essential for combination therapy to elicit complete tumor resolution. NHS-rmIL12 in combination with Entinostat induced only minor, transient toxicity. In the first-in-human phase I trial in patients with advanced malignancies, NHS-IL12 was well tolerated^26^. Entinostat has been proven to be safe and well tolerated in multiple clinical studies^43^. In the studies reported here, Entinostat-induced necrosis facilitated significant NHS-rmIL12 tumor deposition, thereby sustaining synergistic immune stimulatory effects in the TME. This is concordant with a preclinical report of synergistic antitumor effects with NHS-rmIL12 in combination with other treatment modalities, including inducers of immunogenic cell death (ICD) such as radiation and docetaxel chemotherapy^9^. On this basis, other ICD inducers able to promote necrotic cell death and drive adaptive immunity may synergize with NHS-IL12^54^.

Systemically, the sustained presence of elevated cytokines that can drive tumor immune surveillance such as IL-10^44^, GM-CSF, IL-12, and IFNγ was intrinsically associated with early onset and sustained tumor engraftment of highly activated CD8^+^ T cells, including bifunctional IFNγ and TNFα producers. The decreased presence of both M2-like TAMs and Tregs could have further licensed CD8^+^ TILs to drive tumor destruction. It is also likely that Entinostat-driven epigenetic modulation may have contributed to a spectrum of effects^55,56^, as Entinostat has been shown to render carcinoma cells more susceptible to immune killing, including by antigen-specific CD8^+^ T cells^37–39^. Our data suggest that the presence of high levels of IFNγ, GM-CSF, and IFNβ upon combination therapy, gene mapped to CD8^+^ TILs and TAMs, further licensed this cross-talk between innate and adaptive immunity by inducing neutrophils and monocytes to adapt an interferon-responsive, antitumor gene program. In response to TME stimuli such as IFNγ and TNFα, activated macrophages or neutrophils can produce T-cell attracting chemokines, including CXCL9 and CXCL10^7,57^. In addition, the presence of high levels of IL1α and IL1β in the TME produced by macrophages and/or neutrophils activated by combination therapy may have amplified this cooperative mechanism further. Whereas IL1α favors survival and effector function of neutrophils and macrophages, IL1β has been shown to promote myeloid recruitment and trained immunity^46,58^.

Collectively, this resulted in a crescendo in M1 polarization, reprogramming the TME to an inflamed hub where the elevation of monocyte (CCL3, CCL4, CCL5) and CD8 T cell‒attracting chemokines (CXCL9, CXCL10, CCL5) by activated neutrophils and/or macrophages further promoted tumor engraftment of highly functional IFNγ- and TNFα-producing CD8^+^ TILs through a sustained circular loop leading to tumor eradication. This is concordant with reports in which clinical activity of rhIL-12 correlated with the ability to maintain the induction of stimulatory cytokines including IFNγ^12^. *CCL5* and *CXCL9* have been shown to correlate with *CD8A* in the TME across multiple human tumor types^57^. Here, we demonstrated that gene levels of these and other chemokines correlate with increased engraftment of CD8^+^ T cells as well as M1 macrophages across multiple human tumor types, while negatively impacting infiltration of M2 macrophages.

While CD8^+^ T cells are often determinant for tumor eradication, proper signals in the TME are often required to support their effector function, such as IFNγ and IL-12^59^. Recent studies have suggested that M1-like TAMs can contribute to this environment in support of CD8^+^ effector function^60,61^. We speculate that epigenetic reprogramming of systemic monocytes by Entinostat augments their responsiveness to IFNγ and TNFα released in the TME by activated CD8^+^ TILs, leading them to polarize toward M1-like TAMs. Furthermore, the presence of elevated GM-CSF in the TME may have aided M1 polarization and TAM effector function^62^. Using both syngeneic and athymic mice, we showed that in the absence of CD8^+^ T cells, tumor levels of GM-CSF and IFNγ were drastically reduced, with TAMs no longer able to efficiently polarize to M1-like. Moreover, the absence of CD8^+^ T cells negated combination therapy increased levels of additional cytokines (IFNβ, IL1α, IL1β) with neutrophils and macrophages downregulating IFNγ response, and chemokines no longer increased in the TME.

Comparative analysis between MC38 (40% CR) and EMT6 (100% CR) tumor immunomes allowed us to identify some key differences that may help predict response to combination therapy with NHS-rmIL12 and Entinostat. One of the largest differences observed was the sustained activation in CD8^+^ TILs present in EMT6 but lacking in MC38 tumors. Additionally, while Tregs were reduced in both models, MC38 tumors also displayed a significant reduction in CD4^+^ TILs, suggesting a loss of CD4 help. Whereas a consistent repolarization of TAMs towards a M1-like phenotype was observed in both models, the magnitude (M1/M2 ratios) of this effect was significantly higher in the EMT6 TME.

In conclusion, using comprehensive TME single-cell transcriptome, proteome, and immune cell analysis, we demonstrated that combination therapy elicits significant and sustained antitumor efficacy and complete responses by shifting the tumor immunome to a functionally inflamed landscape, where the concerted actions of highly active CD8^+^ T cells, neutrophils, and M1-like antitumor macrophages leads to complete tumor eradication. Notably, based on these findings, we identified a five-gene signature associated with overall survival across a spectrum of malignancies, including poorly inflamed tumor types such as breast cancer. These findings thus provide a rationale for combining NHS-IL12 with Entinostat in the clinical setting.

## Methods

### Reagents

Under Cooperative Research and Development Agreements with the National Cancer Institute (NCI), Entinostat and NHS-rmIL12 were provided by Syndax and EMD Serono, respectively. Entinostat was formulated in a low-fat diet with 35% sucrose for a target murine dose of 6 mg/kg/day (Research Diets). Recombinant murine IL-12 (rmIL12) was obtained from Peprotech. NHS-rmIL12-alexafluor 647 (AF647) was generated by conjugating AF647 to NHS-rmIL12 using the SAIVI^TM^ Alexa Fluor^TM^ 647 Antibody/Protein 1 mg—Labeling Kit (ThermoFisher) following the manufacturer’s instructions. Labeling was confirmed by flow cytometry and SDS-PAGE /fluorescent imaging. CD8 (2.43) depletion antibody was obtained from BioXcell.

### Tumor cell lines

Murine breast (EMT6, CRL-2755) and colon (CT26, CRL-2638) carcinoma cells obtained from American Type Culture Collection. Murine breast TS/A tumor cells^51,63^ were a kind gift from Dr. Donald Buchsbaum (University of Alabama at Birmingham) with permission from Dr. Pier-Luigi Lollini (Univ. Bologna, Italy). EMT6 and CT26 cell lines were obtained with the certificate from the vendor. TS/A and MC38 cell lines were not authenticated. All cell lines were cultured according to the provider’s instructions. Murine colon carcinoma MC38 cells are as described^64^. All cell lines were free of *mycoplasma* as determined by MycoAlert Mycoplasma Detection Kit (Lonza) and were used at low passage numbers.

### Animals

Six- to 8-week-old female Balb/c, nu/nu, and CEA-Tg mice were obtained from the NCI Frederick Cancer Research Facility (Frederick, MD). Animals were co-housed in microisolator cages under pathogen-free conditions and a 12 h:12 h light/dark cycle, in rooms at 72 °F ± 2 °F and 30-70% relative humidity, in an Association for Assessment and Accreditation of Laboratory Animal Care-accredited animal facility of the National Institutes of Health (NIH). For select studies, mice were euthanized by cervical dislocation. All studies were reviewed and approved by the NIH Intitutional Animal Care and Use Committee. We have complied with all relevant ethical regulations for animal testing and research.

### Murine tumor studies

On day 0, MC38 (3 × 10^5^) or CT26 (1 × 10^6^) were implanted subcutaneously (s.c.) into the right flank of CEA-Tg or Balb/c female mice, respectively. EMT6 (2.5 × 10^5^) and TS/A (1 × 10^5^) were orthotopically implanted into the mammary fat pad of female Balb/c or nu/nu mice. Seven to 11 days after, mice were randomized to receive control or Entinostat diet. On day 14–16, mice were re-randomized within each diet cohort to receive diet alone or combined with NHS-rmIL12 (2 μg, s.c.) administered every other day for 3 doses. In select studies, animals were administered rmIL12 (1.1 μg, s.c.) similar in molarity to NHS-rmIL12 dosing (2 μg). The NHS-rmIL12-AF647 was given at a dose of 50 μg (s.c.). Animals were removed from special diet 4 days after the last dose of NHS-rmIL12. In select studies, special diet was initiated at the time of first NHS-rmIL12 dosing or continued until the end of the experiment. At least 1 month after tumor resolution, cured and naïve control mice were rechallenged with the same tumor cells alone or in addition to different tumor cells injected in the opposing mammary fat pad. Tumor volume was measured twice weekly and calculated by (length^2^ × width)/2. Survival was monitored. Treatment-related toxicity was assessed in EMT6 tumor-bearing mice by monitoring animal body weight, serum chemistry, and organ histopathology at designated time points.

#### Depletion studies

CD8 (2.43) depletion antibody was administered intraperitoneally (100 μg) to EMT6 tumor-bearing mice on days 14, 15, and 16 post-tumor implant and then once weekly for the duration of the experiment.

#### Necrosis assessment and NHS-rmIL12 tumor localization

MC38 and EMT6 tumors were harvested after 14 days on Entinostat feed, preserved in Z-fix (Anatech) and paraffin embedded. Tumor sections were stained with H&E using standard procedures (HistoServ, Inc.). All tumor sections were imaged with an AxioScan Z1 Slide Scanner (Zeiss). Necrosis was quantified using Zen Blue v 2 by tracing areas of necrosis versus total tumor area. NHS-rmIL12-AF647 was imaged in resected tumors using the Odyssey CLx (LI-COR) and the fluorescent area was quantified using ImageJ (NIH). To quantify NHS-rmIL12 in serum and TME supernatant, the anti-human IgG portion of NHS-rmIL12 was detected using Human IgG ELISA Kit (Sigma-Aldrich) following the manufacturer’s protocol. To generate tumor supernatant, tumors were homogenized in PBS using the gentleMACS Dissociator according to the manufacturer’s instructions (Miltenyi Biotec). The supernatant was removed and stored at −80 °C until use.

### Ex vivo analysis

#### Preparation of single-cell suspensions

Single-cell suspensions from individual tumor and spleens were prepared using standard procedures. Briefly, spleens were harvested, smashed using 120 μM nylon sheets, filtered through a 70 μM filter, and subjected to ACK lysis. Tumors were harvested, cut into 2 mm pieces and processed using the gentleMACS Dissociator according to the manufacturer’s instructions (Miltenyi Biotec). After digestion, single cell suspensions were filtered through a 70 μM filter. Cell counts were performed using 123 count eBeads (ThermoFisher).

#### Flow cytometric analysis

Staining of immune cells (~1 × 10^6^) for flow cytometry was performed using the Cytofix/Cytoperm Kit (BD Biosciences) according to the manufacturer’s instructions. Antibodies and dilutions used are listed in Supplementary Table 3 with matched isotypes obtained from the aforementioned manufacturers. Data were acquired on a BD FACSVerse with BD FACSuite software v1.0.6.5320, or LSRII Fortessa flow cytometer with FACS Diva v 9.0 software (BD Biosciences) and analyzed with FlowJo Analysis Software v9.9.6 (BD Biosciences). Cell populations were identified using gating strategies shown in Supplementary Table 4 and Supplementary Fig. 9. All frequencies of phenotypic proteins were generated by subtracting the frequency of respective isotype, typically set between 1 and 5%.

#### Immune cell functional assays

Lymphocytes (~1 × 10^6^) from tumor single-cell suspensions were either unstimulated or stimulated in vitro with 1 μg/mL anti-CD3 (2C11) and anti-CD28 (37.51) for 4 h in the presence of 2 μg/mL Golgi Plug (BD Biosciences). The frequency of cells expressing intracellular IFNɣ and TNFα were quantified by flow cytometry. Splenocyte IFNɣ responses to tumor cells were monitored using Mouse IFNɣ ELISPOT kit (BD Biosciences) according to the manufacturer’s protocol. Briefly, 1 × 10^5^ splenocytes were co-cultured for 24 h without tumor cells or with 4 × 10^4^ EMT6 or TS/A tumor cells. Spot-forming cells (SFC) were quantified with an ImmunoSpot analyzer using the Smart Count ImmunoSpot SC Suite v 2.6.1 software (Cellular Technology, Ltd.).

#### Detection and quantification of serum cytokines and tumor chemokines

Serum cytokines were quantified using the V-PLEX Proinflammatory Panel I Mouse Kit and MESO QuickPlex SQ 120 (Meso Scale Diagnostics, LLC). Limits of detection (pg/ml) were: IFNγ: 0.04, IL-12p70: 9.95, IL-10: 0.95, TNFα: 0.13. TME supernatants were generated as described above. Tumor chemokines and cytokines were detected using the LEGENDplex Mouse 13-Plex Proinflammatory Chemokine Panel and Mouse 13-plex Inflammation Panel (Biolegend), respectively. Data were collected on the BD FACSVerse flow cytometer (Beckton Dickinson) and analyzed with FlowJo FACS Analysis Software v9.9.6 (BD Biosciences).

#### Single-cell RNA sequencing and data analysis

Single-cell suspensions from EMT6 tumors obtained 2 days after the last NHS-rmIL12 (2 μg, s.c.) were prepared as described above. Single-cell RNA sequencing with 10X Genomics Cellranger v 4.0.0 was performed on pooled tumor-infiltrating CD45^+^ cells, isolated by magnetic selection from individual tumors using CD45 (TILs) MicroBeads, according to the manufacturer’s instructions (Miltenyi Biotec). Data analyses were performed as described in the Supplementary Methods.

### Statistics

Statistical analyses were performed on data from three or more biologically independent experimental replicates. Statistical analysis was conducted in GraphPad Prism 7 (GraphPad Software) and reported in the figures and corresponding figure legends. Statistical significance was set at *p* < 0.05. Grey = *p* < 0.05; red = *p* < 0.01; blue = *p* < 0.001; black = *p* < 0.0001.

Additional materials and methods are described in the Supplementary Methods.

### Reporting summary

Further information on research design is available in the Nature Research Reporting Summary linked to this article.

## Supplementary information

Supplementary Information

Description of Additional Supplementary Files

Supplementary Data 1

Supplementary Data 2

Supplementary Data 3

Supplementary Data 4

Supplementary Data 5

Supplementary Data 6

Reporting Summary

## Data Availability

ScRNASeq data have been deposited in GEO, #GSE171273. Human gene transcript TCGA (https://portal.gdc.cancer.gov) and GTEx (http://gtexportal.org) datasets are identified in figure legends and were accessed through TIMER2.0 (http://timer.comp-genomics.org) and/or GEPIA2 (http://gepia2.cancer-pku.cn). Additional source data are provided with this paper. All relevant data are available from the authors upon request. Source data are provided with this paper.

## Source data

Source Data

## Code availability

All scRNASeq data were analyzed using custom code written in R based on open source software. All scripts used have been deposited in github^45^:

https://github.com/CCBR/Antitumor-activity-of-entinostat-plus-NHS-IL12; 10.5281/zenodo.4891285.
