# Supplementary material for: Tumour-targeted interleukin-12 and entinostat combination therapy improves cancer survival by reprogramming the tumour immune cell landscape
Source: Nat Commun. 2021 Aug 26;12:5151. doi: 10.1038/s41467-021-25393-x (PMC8390765; doi:10.1038/s41467-021-25393-x)
Supplement: Supplementary file 1 — Supplementary Information [file 41467_2021_25393_MOESM1_ESM.pdf]

Tumour-targeted Interleukin-12 and Entinostat Combination Therapy Improves Cancer Survival by Reprogramming the Tumour Immune Cell Landscape
by Hicks et al.

SUPPLEMENTARY FIGURES 1-9
SUPPLEMENTARY TABLES 1-5
SUPPLEMENTARY METHODS

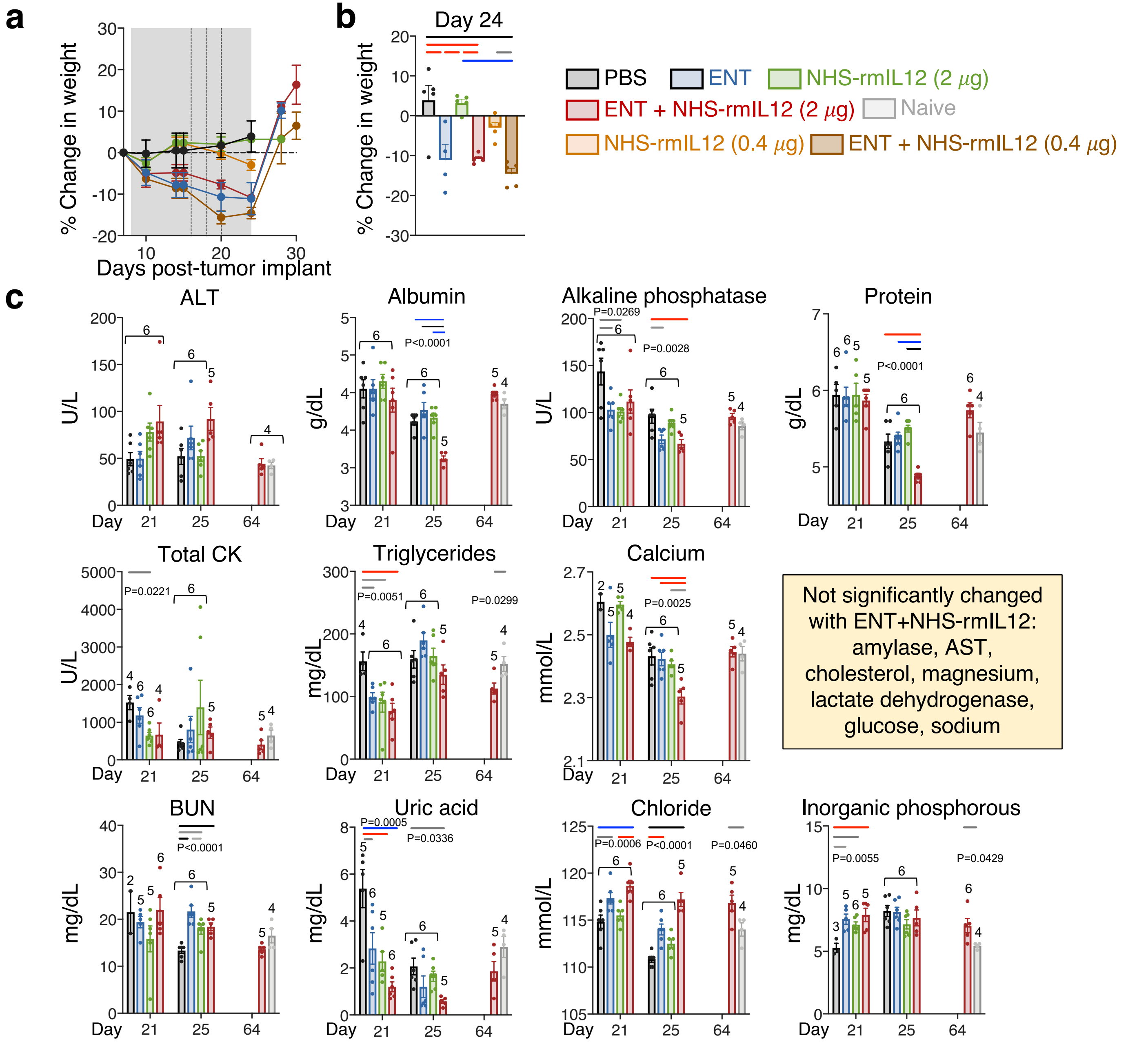

d

Organ	PBS D21	ENT D21	NHS D21	ENT+NHS D21	ENT+NHS D50	Naive
**Lung**	3/3 pneumonitis 2/3 moderate 1/3 mild	3/3 pneumonitis 3/3 severe	3/3 pneumonitis 2/3 moderate 1/3 mild	3/3 pneumonitis 1/3 moderate 2/3 mild	3/3 pneumonitis 3/3 moderate	1/2 normal 1/2 pneumonitis 1/2 moderate
**Duodenum**	1/3 normal 2/3 epithelial necrosis	3/3 epithelial necrosis	1/3 normal 2/3 epithelial necrosis	1/3 normal 2/3 epithelial necrosis	3/3 epithelial necrosis	1/2 normal 1/2 epithelial necrosis
**Kidney**	3/3 normal	3/3 normal	3/3 normal	2/3 normal 1/3 nephritis	3/3 normal	2/2 normal
**Liver**	3/3 normal	3/3 normal	3/3 hepatitis 3/3 moderate	1/3 normal 1/3 moderate hepatitis 1/3 mild cholangitis	3/3 normal	2/2 normal

All mice had normal hearts and brains

Supplementary Figure 1: Analysis of toxicity of ENT+NHS-rmIL12 by monitoring body weight change, serum chemistry, and organ histopathology. EMT6 tumor-bearing mice (n=5/group) were treated as in Fig. 1a. **(a)** Change in body weight over time with treatment and **(b)** at day 24 ($P < 0.0001$).**(c)** Serum chemistry analysis of EMT6 tumor-bearing mice at days 21 and 25, and cured mice at day 64, and naïve mice (n=4). **(d)** Organ histopathology of EMT6 tumor-bearing mice at day 21, cured mice at day 50, and naïve mice. Graphs depict individual values, and/or mean±SEM. Inset numbers denote number of independent samples/group analyzed. One-way ANOVA with Tukey's multiple comparisons test used for day-specific comparisons, except two-tailed unpaired t test for all day 64 data (panel c). Grey=$p<0.05$, red=$p<0.01$, blue=$p<0.001$, black=$p<0.0001$. Data are from single independent experiments. ALT, alanine aminotransferase; AST, aspartate aminotransferase; ENT, entinostat.

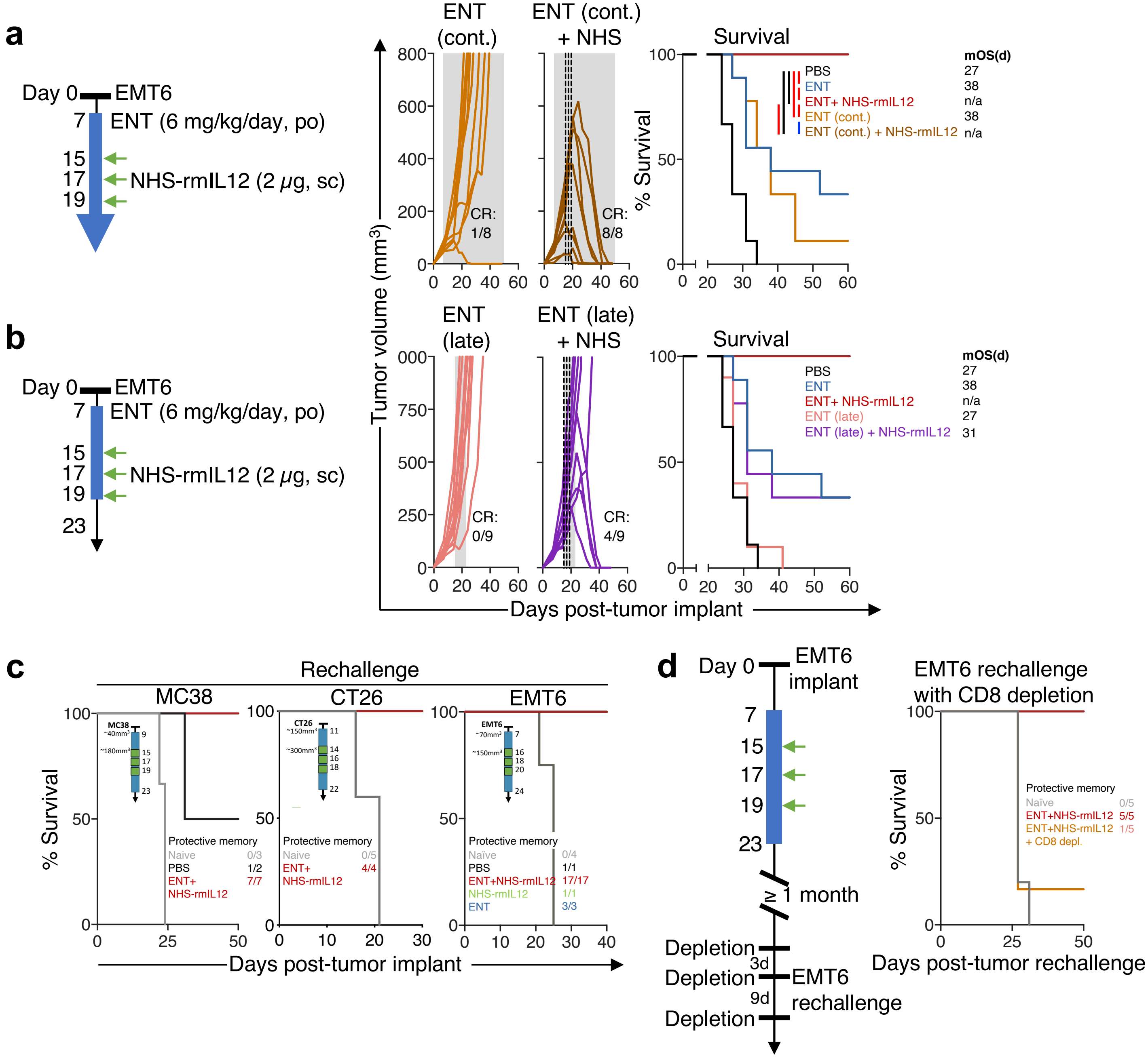

Supplementary Figure 2: Effect of entinostat (ENT) treatment schedule on the anti-tumor efficacy of combination therapy. Treatment schematic, individual tumor growth curves, and survival for EMT6 tumor-bearing mice treated with **(a)** continuous (cont.) entinostat treatment starting at day 7 post-tumor implant. Treatment schematic, individual tumor growth curves, and survival for EMT6 tumor-bearing mice treated with **(b)** delayed (late) onset of entinostat administration to day 15 post tumor implant. Gray shaded areas indicate duration of ENT treatment and dashed lines depict NHS-rmIL12 (NHS) doses. Tables show median overall survival (mOS) in days . * indicates data are one of the three repeats of Fig 1b.

ENT+NHS-rmIL12 produces a tumor specific and CD8-dependent protective memory. (c) Mice cured in experiments shown in **Figure 1** were rechallenged at least 1 month post-curing with same tumor type and monitored for tumor growth. Graphs show survival data and insets denote specific treatment schedules utilized for each model in **Figure 1**. Tables below indicate the number of mice that had protective memory. **(d)** Cured mice were depleted or not of $CD8^{+}$ T cells as indicated in the schematic prior to rechallenge with EMT6 tumors. Graph shows survival and table below indicates the number of mice that had protective memory. Data are from single independent experiments. ENT, entinostat.

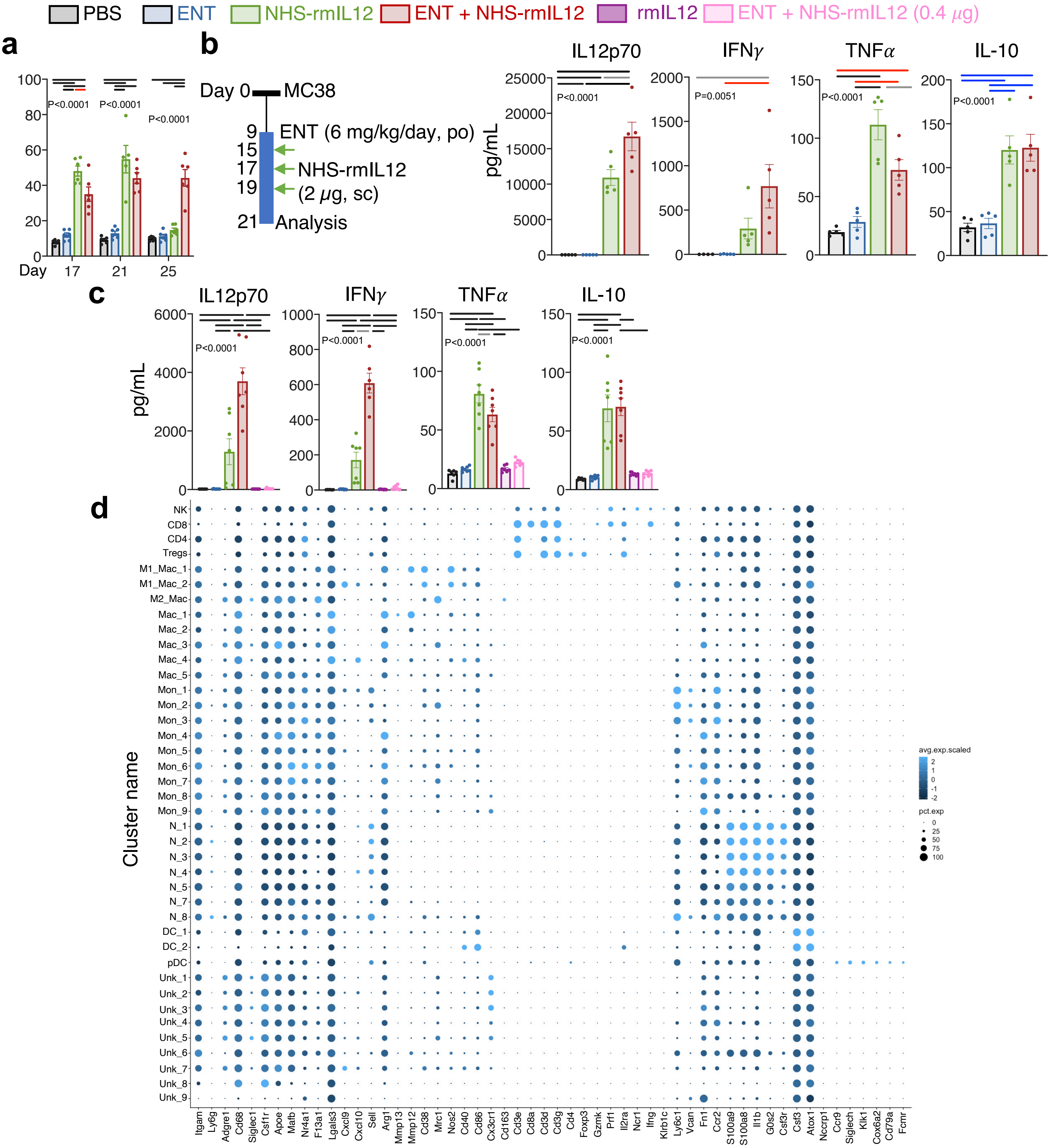

Supplementary Figure 3: ENT+NHS-rmIL12 promotes higher levels of proinflammatory serum cytokines than ENT+rmIL-12 in EMT6 and has similar effects in the MC38 model. (a) Serum levels of IL-10 (n=6 mice/group) at designated days post-tumor implant from EMT6 tumor-bearing mice treated as in Fig. 1a. **(b)** MC38 tumor-bearing mice treatment schematic and serum cytokine levels (n=5 mice/group) at day 21 post-tumor implant. **(c)** Serum cytokine levels (n=5 mice/group) in EMT6 tumor-bearing mice 2 days after last NHS-rmIL12 dose from experiment in Fig. 1b. All graphs show mean±SEM with values from individual mice. One-way ANOVA with Tukey's multiple comparisons test used for day-specific comparisons. Grey = p<0.05, red = p<0.01, blue = p<0.001, black = p<0.0001. **(d)** Average expression scaled of selected genes used in the identification of each immune gene cluster by scRNAseq in tumor CD45+ cells from EMT6-treated mice on day 21 as in Fig. 1a. Data from single independent experiments.

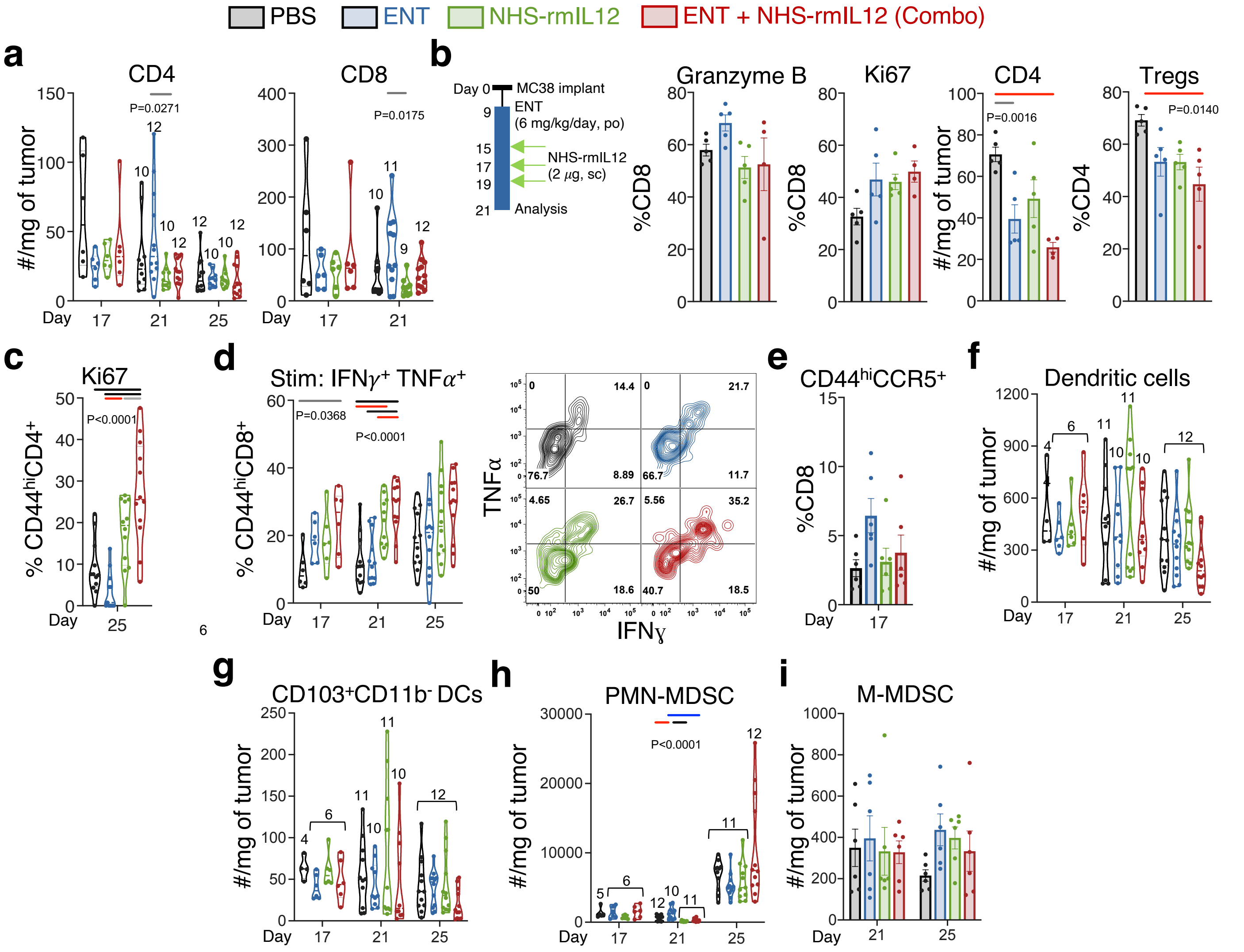

Supplementary Figure 4: Other effects of ENT+NHS-rmIL12 on tumor immunome. EMT6 tumor-bearing mice were treated as in Fig. 1a. Quantification of **(a)** CD4^{+}FoxP3^{-} and CD8^{+} TILs on days 17 (n=6), 21, and 25 post-tumor implant. **(b)** MC38 tumor-bearing mice were treated as per schematic. Graphs show expression of activation markers granzyme B and Ki67 on CD8^{+} TIL (n=5, except combo, n=4) and quantification of CD4^{+}FoxP3^{-} TILs (n=5, except combo, n=4) and CD4^{+}FoxP3^{+} Treg TILs (n=5) on day 21. **(c)** Expression of activation marker Ki67 on CD4^{+}FoxP3^{-} TIL from EMT6 tumors (n=12, except ENT, n=11) treated as in Fig. 1a. **(d)** Quantification of bifunctional, IFNγ/TNFα producing, CD44^{hi}CD8^{+} TILs from EMT6 tumors treated as in Fig. 1a and examined by flow cytometry on days 17 (n=6), 21 (n=12) and 25 (n=12) post-tumor implant upon stimulation *ex vivo* with αCD3/αCD28. Representative flow cytometric analysis on day 21. Expression of **(e)** CD44^{hi}CCR5^{+} on splenic CD8^{+} T cells on day 17 post-EMT6 tumor implant in mice treated as in Fig. 1a, n=6. Quantification of **(f)** dendritic cells (DC), **(g)** CD103^{+}CD11b^{-} DCs, **(h)** PMN-MDSC/Neutrophil, and **(i)** M-MDSC (n=6) tumor infiltration on designated days post-tumor implant. Bar graphs show mean ± SEM with values from individual mice. Truncated violin plots show values from individual mice with contours denoting kernel density distributions; dashed line, median; and dotted line, interquartile range. Inset numbers denote number of independent samples/group. One-way ANOVA with Tukey's multiple comparisons test used for day-specific comparisons. Grey = $p<0.05$, red = $p<0.01$, blue = $p<0.001$, black = $p<0.0001$. Flow cytometry data from day 17, and M-MDSC is representative of one independent experiment, with all other data pooled from two experiments conducted independently with similar results. ENT, entinostat; M-MDSC, monocytic myeloid-derived suppressor cell; PMN-MDSC, polymorphonuclear MDSC. TIL, tumor-infiltrating lymphocytes.

Dendritic cells were gated on Live>Singlets>Live/Dead^{neg}>CD45^{+}>F4/80^{neg}>Ly6g^{neg}> CD11c^{+}IA/IE^{+}.
PMN-MDSC were gated on Live>Singlets>Live/Dead^{neg}>CD45^{+}>CD11b^{+}>Ly6C^{med}>Ly6G^{+}.
M-MDSC were gated on Live>Singlets>Live/Dead^{neg}>CD45^{+}>CD11b^{+}>Ly6G^{neg}->Ly6C^{+}.

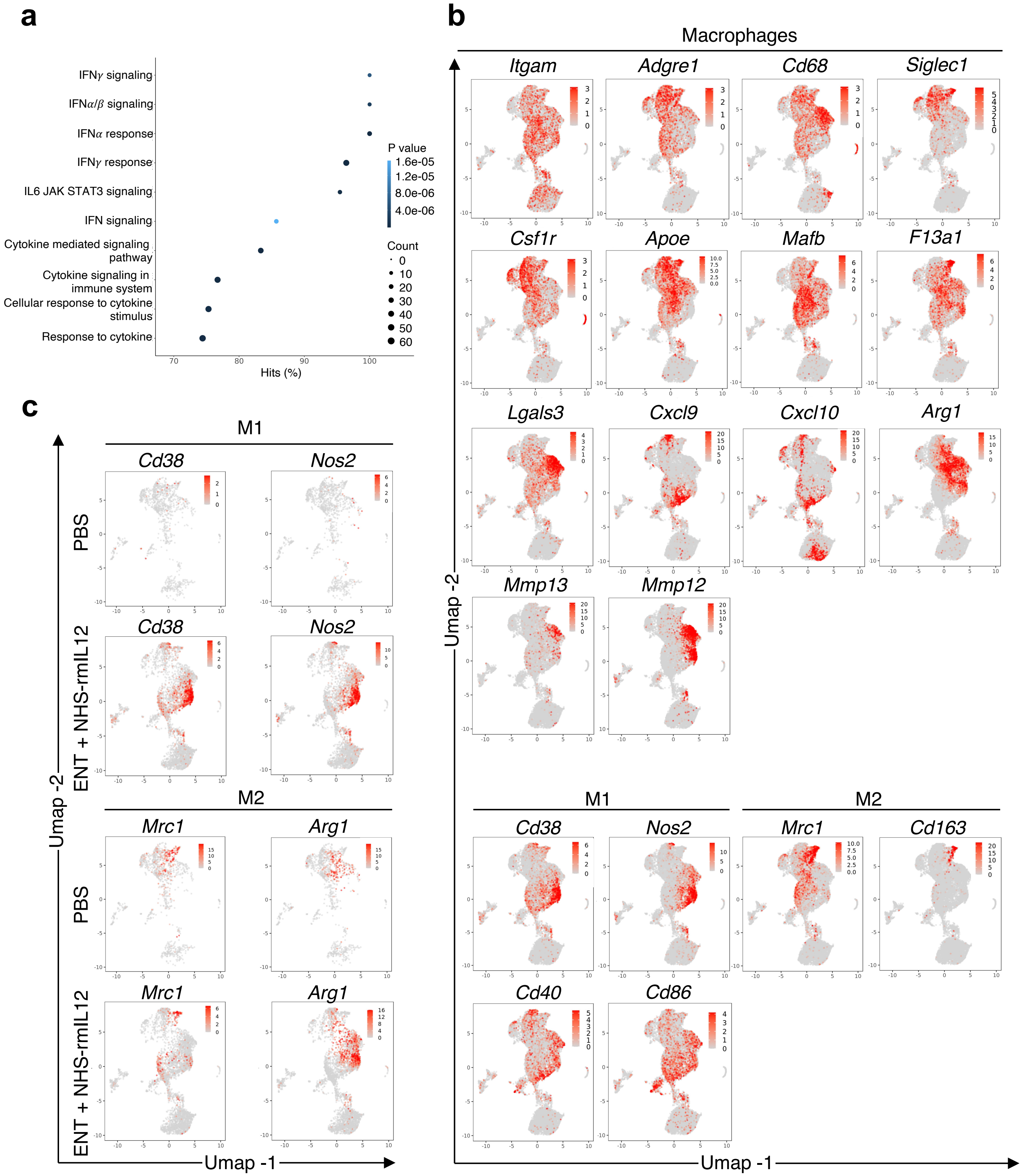

Supplementary Figure 5: EMT6 tumor-bearing mice were treated as in Fig. 1a. **(a)** Top 10 upregulated gene pathways in EMT6 tumor-infiltrated monocyte gene clusters identified by scRNAseq in response to combination therapy on day 21 post-tumor implant vs. PBS controls. UMAPs show expression mapping of **(b)** macrophage-associated genes, including those associated with M1 (upper box) or M2 (lower box) TAMs, or **(c)** effect of combination therapy on expression of M1 and M2-associated genes. Data from one single experiment. ENT, entinostat; TAMs, tumor-associated macrophages.

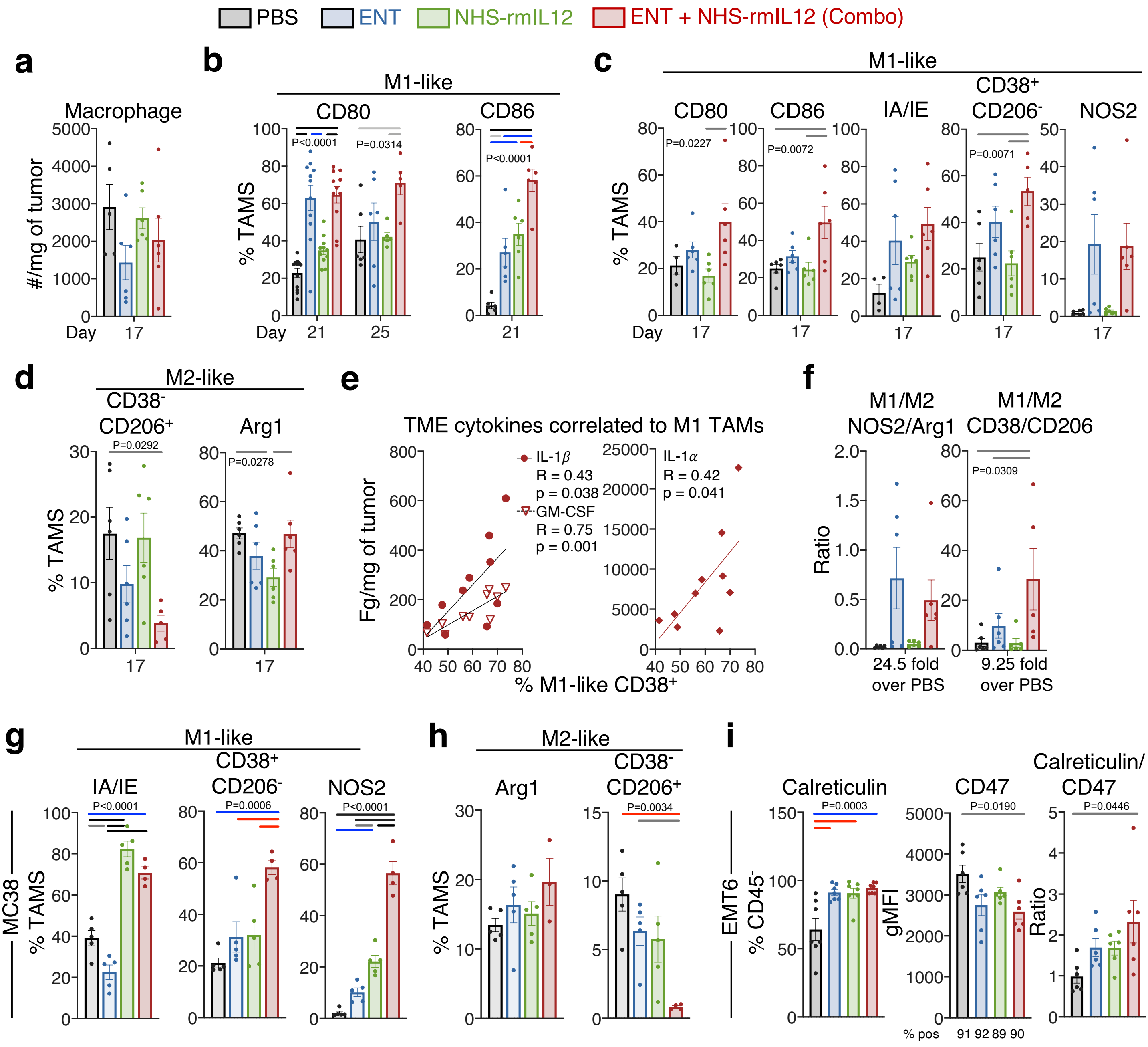

Supplementary Figure 6: Combination therapy shifts the balance of TAMs from M2-like towards M1-like on day 17. EMT6 tumor-bearing mice were treated as in Fig. 1a. **(a)** Quantification of TAM on day 17 post-tumor implant, n=6 mice/group, data from one independent experiment. **(b)** Expression of M1-like markers CD80 and CD86 on TAMs on days 21 and 25 post-tumor implant. Day 21 CD80 data was pooled from two independent experiments with similar results, n=12 mice/group. Day 25 CD80 and day 21 CD86 data is from one independent experiment, n=6 mice/group. Expression of **(c)** M1-like markers and **(d)** M2-like markers on TAMs on day 17 post-implant. Data from one independent experiment, n=6 mice/group. **(e)** Correlation between designated cytokine levels and tumor volume change between days 14 and 18 for each combination-treated mouse. For correlations, Pearson's correlation coefficient was performed. Data from one independent experiment. **(f)** M1/M2 ratios with indicated markers on day 17 post-implant from data in panels c-d, n=6 mice/group. MC38 tumor-bearing mice were treated as in Supplemental Figure 4b. Expression of designated **(g)** M1-like and **(h)** M2-like markers on TAMs at day 21 post-tumor implant. Data from one independent experiment, n=6 mice/group. **(i)** EMT6 tumor-bearing mice were treated as in Fig. 1a. Expression of cell-surface calreticulin, CD47, and the ratio of the two on non-immune cells in the tumor on day 17 post-tumor implant. Data from one independent experiment, n=6 mice. All graphs show mean±SEM and/or values from individual mice. One-way ANOVA with Tukey's multiple comparisons test used for comparisons. Grey = p<0.05, red = p<0.01, blue = p<0.001, black = p<0.0001. ENT, entinostat; TAM, tumor-associated macrophage. TME, tumor microenvironment.

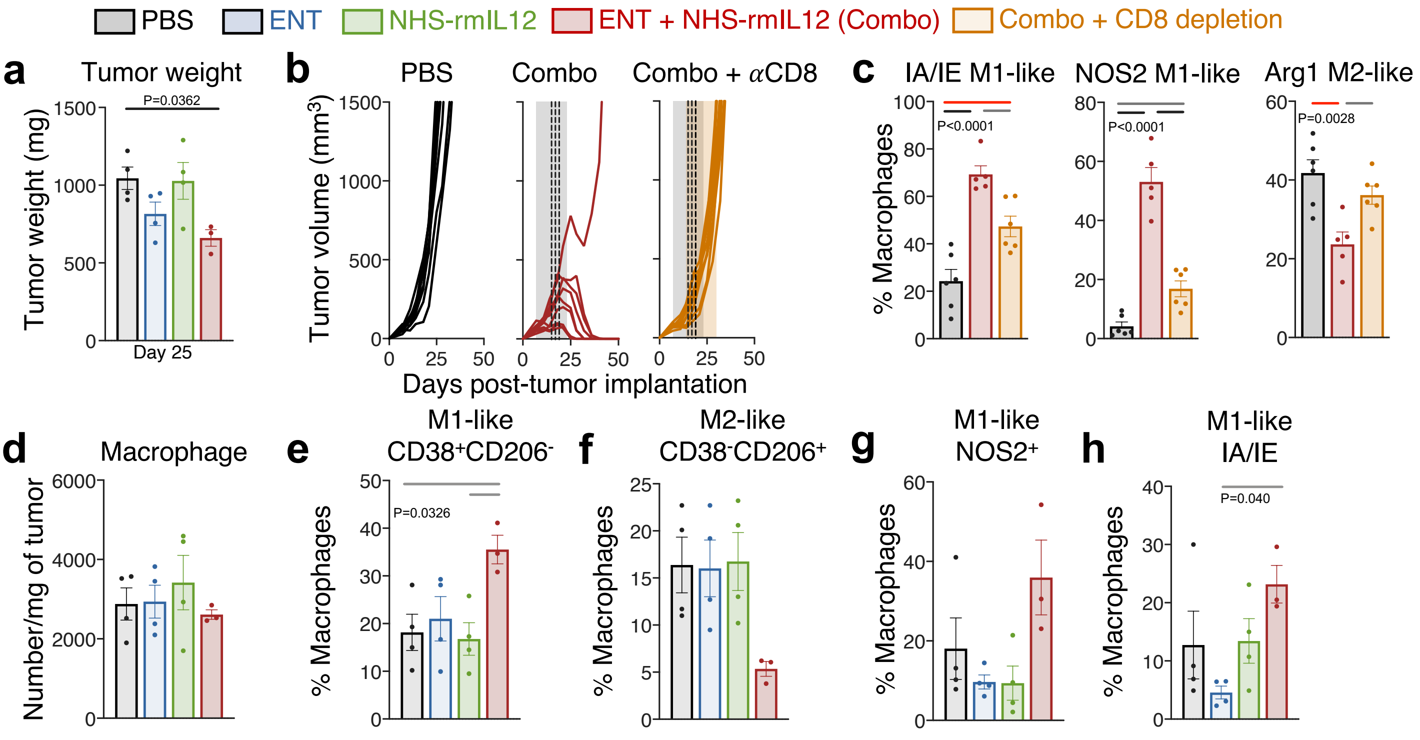

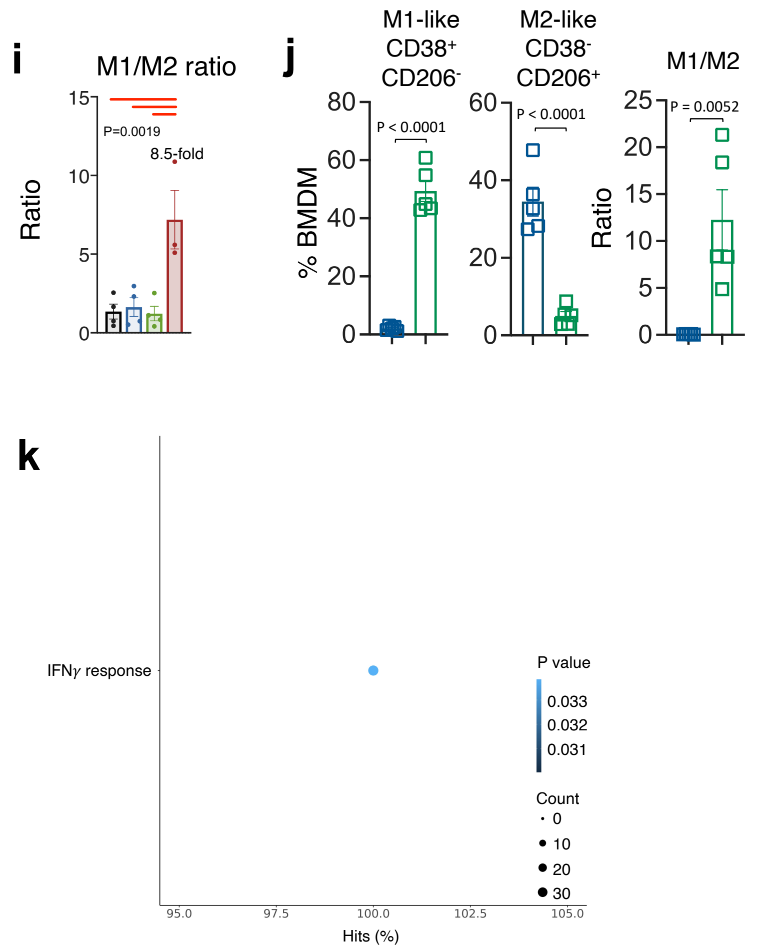

Supplementary Figure 7: ENT+NHS-rmIL12 repolarizes TAMs in EMT6 tumor-bearing nude mice to a lesser extent than observed in syngeneic mice. **(a)** EMT6 tumor-bearing nude mice were treated as indicated in Fig. 7a. Tumor weight (n=4, except combo, n=3) at time of sacrifice on day 25 post tumor implant. **(b)** Individual tumor growth corresponding to data in Fig. 7b&c. Expression of **(c)** M1-like markers IA/IE and NOS2 as well as M2-like marker Arg1 corresponding to Fig. 6j (n=6, except combo, n=5). In nude mice (n=4, except combo, n=3), **(d)** TAM infiltration, **(e)** M1-like and **(f)** M2-like TAMs denoted by CD38 and CD206 expression. **(g&h)** Expression of additional M1-like markers NOS2 and IA/IE in nude mice (n=4, except combo, n=3). **(i)** M1 to M2 TAM ratio in nude mice (n=4, except combo, n=3). **(j)** Expression and ratio of CD38 and CD206 on BMDM (n=5) from PBS-treated EMT6 tumor-bearing Balb/c mice incubated with nothing (M0) or IFNγ/TNFα. All graphs show mean±SEM and/or values from individual mice. **(k)** Gene pathways downregulated in neutrophil clusters identified by scRNAseq in EMT6 tumors from CD8-depleted mice treated with combination therapy. One-way ANOVA with Tukey's multiple comparisons test, except unpaired two-tailed t test **(j)**. Grey = p<0.05, red = p<0.01, blue = p<0.001, black = p<0.0001. Data are representative of one independent experiment except **(c&d)** are representative from 2 independent experiments with similar results. BMDM, bone marrow-derived macrophages; ENT, entinostat; NHS, NHS-rmIL12; TAM, tumor-associated macrophage.

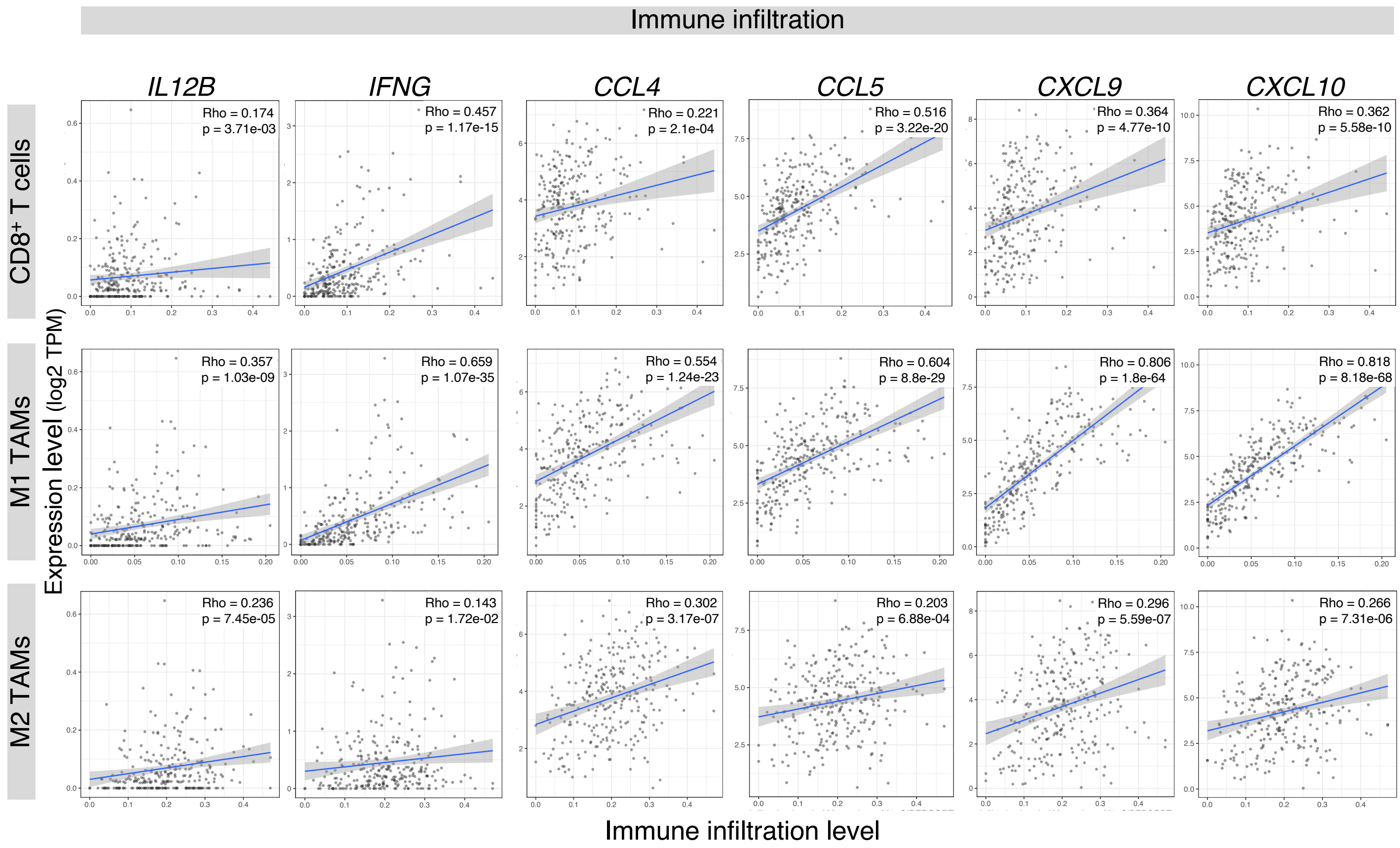

Supplementary Figure 8: Correlations between murine studies and COAD human cancers. Dot plot of two-tailed Spearman's correlation between genes of key immune markers responsible for the anti-tumor efficacy of NHS-rmIL12+entinostat treatment in tumor-bearing mice, and immune infiltration observed in a human colon adenocarcinoma (COAD) TCGA patient dataset (n=458). Data was generated using TIMER2.0. Line represents best-fitting regression line with 95% confidence interval (grey shading). Spearman Rho > 0 indicates positive correlation; Spearman Rho < 0 indicates negative correlation. TAM, tumor-associated macrophage.

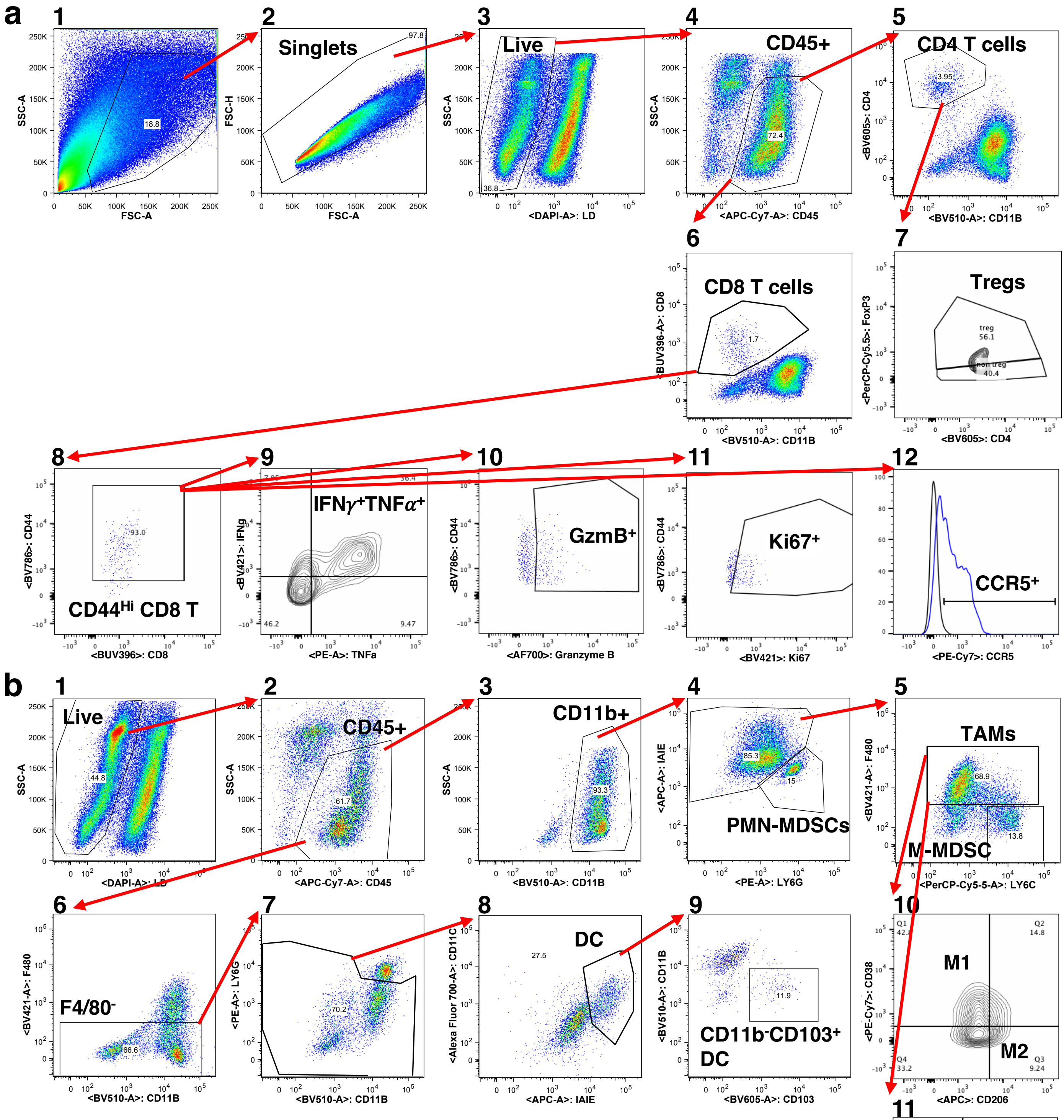

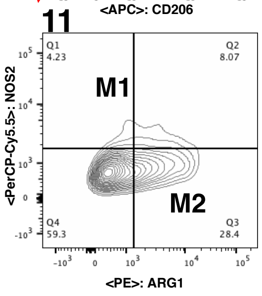

Supplementary Figure 9: Gating strategies used to identify murine immune subsets by flow cytometry. (a) Gating strategy used to identify CD8^{+} T cells (panel 6) and their subsequent phenotype (panel 9-12), CD4^{+} T cells (panel 7), and regulatory CD4^{+}FoxP3^{+} T cells (Tregs, panel 7), pertaining data in Figure 3d-j, Figure 4e-i, Figure 5c, and Figure 7g. **(b)** Gating strategy used to identify PMN-MDSC/Neutrophils (panel 4), macrophages (panel 5), M1- and M2-like macrophages (panel 10 and 11), M-MDSCs (panel 5), dendritic cells (DCs, panel 8), and CD103^{+} DCs (panel 9), pertaining to data in Figure 6e-j, m-o, and Figure 7h-j. GzmB, granzyme B; M-MDSC, monocytic myeloid-derived suppressor cells; PMN-MDSC, polymorphonuclear myeloid-derived suppressor cells; TAM, tumor-associated macrophage.

Supplementary Table 1: Marker genes used for cell type identity by scRNAseq

CD8_T	CD4_T	Tregs	Macrophages	M1	M2	Neutrophils	Monocytes	cDCs	pDC	B_cells	NKs
Cd3e pos	*Cd3e pos*	*Cd3e pos*	*Itgam high*	*Cd38 pos*	*Cd38 neg*	*Ly6g pos*	*Itgam pos*	*Cst3 pos*	*Ccr9 pos*	*Cd79a pos*	*Cd3e neg*
Cd8a pos	*Cd4 pos*	*Cd4 pos*	*Ly6g neg*	*Mrc1 neg*	*Mrc1 pos*	*Ly6c1 low*	*Ly6c1 pos*	*Atox1 pos*	*Siglech pos*	*Fcmr pos*	*Ncr1 pos*
Cd3d pos	*Foxp3 neg*	*Foxp3 pos*	*Adgre1 pos*	*Nos2 pos*	*Cd163 pos*	*Adgre1 neg*	*Ly6g low*	*Nccrp1 pos*	*Cox6a2 pos*		*Ifng pos*
Cd3g pos	*Cd3d pos*	*Cd3d pos*	*Cd68 pos*	*Sell low*		*S100a9 pos*	*Vcan pos*		*Klk1 pos*		*Klrb1c pos*
Cd4 neg	*Cd3g pos*	*Cd8a neg*	*Siglec1 pos*	*Cd40 pos*		*S100a8 pos*	*Fn1 pos*				
Foxp3 neg	*Cd8a neg*	*Il2ra pos*	*Csf1r pos*	*Cd86 pos*		*Il1b pos*	*Ccr2 pos*				
Gzmk pos	*Prf1*		*Apoe pos*			*G0s2 pos*	*Csf1r pos*				
			Mafb pos			*Csf3r pos*	*Mafb high*				
			Cx3cr1 pos				*Nr4a1 pos*				
			Nr4a1 neg				*Lgals3 pos*				
			F13a1 pos								
			Lgals3 pos								
			Cxcl9 pos								
			Cxcl10 pos								
			Sell low								
			Arg1 pos								
			Mmp13 pos								
			Mmp12 pos								

Supplementary Table 2: Number of cell types within each cluster

Cell Type	B cells	NK	CD8_T	CD4_T	Treg	M1	M2	Macrophages	Monocytes	Neutrophils	DCs	pDC	Unknown
NK	0	73	44	25	1	3	0	6	7	13	1	0	34
CD8	0	7	124	40	1	0	0	0	1	0	1	0	0
CD4	0	0	10	99	8	0	0	1	13	4	2	3	36
Tregs	0	0	1	73	52	0	0	1	4	5	1	0	4
M1_Mac_1*	0	0	1	0	0	185	0	236	49	3	1	0	176
M1_Mac_2*	0	0	0	0	0	73	0	79	104	1	2	0	289
M2_Mac*	0	0	0	1	0	1	58	169	52	13	1	0	137
Mac_1	0	0	0	0	0	19	0	757	16	0	0	0	41
Mac_2	0	0	0	0	0	12	1	184	17	0	1	0	178
Mac_3	0	0	0	0	0	0	11	261	58	0	0	0	39
Mac_4	0	0	0	0	0	21	0	121	25	5	1	0	63
Mac_5	0	1	0	0	0	0	0	15	10	0	0	0	13
Mon_1	0	0	0	0	0	2	0	26	391	0	0	0	306
Mon_2	0	0	0	1	0	0	2	52	291	1	1	0	196
Mon_3	0	0	0	0	0	0	0	4	240	0	0	0	117
Mon_4	0	0	0	0	0	0	0	184	234	1	0	0	29
Mon_5	0	0	0	0	0	18	1	209	266	0	1	0	182
Mon_6	0	0	0	0	0	3	0	103	258	2	1	0	29
Mon_7	0	0	0	0	0	1	0	82	217	0	0	0	51
Mon_8	0	0	0	0	0	3	4	79	98	42	2	0	91
Mon_9	0	0	0	0	0	0	0	42	106	0	0	0	39
N_1	1	0	0	0	0	0	0	4	9	770	0	0	2
N_2	0	0	0	0	0	0	0	2	2	730	0	0	7
N_3	0	1	0	0	0	0	0	2	8	702	0	0	0
N_4	0	0	0	0	0	0	0	5	4	441	0	1	12
N_5	0	1	0	0	0	0	0	13	3	497	0	0	75
N_7	0	0	0	0	0	2	1	8	25	39	0	0	34
N_8	0	0	0	0	0	0	0	6	64	95	0	0	20
DC_1	0	0	0	0	0	0	0	1	13	7	234	0	118
DC_2	0	0	0	0	0	0	0	0	0	0	30	0	3
pDC	9	0	0	0	0	0	0	0	0	0	1	12	10
Unk_1	0	0	0	0	0	1	5	133	62	2	0	0	330
Unk_2	0	0	0	2	0	1	1	57	51	4	2	1	400
Unk_3	0	0	0	0	0	2	1	81	76	2	0	0	134
Unk_4	1	0	0	0	0	0	2	27	92	1	0	1	160
Unk_5	0	0	0	0	0	3	7	137	66	0	0	0	202
Unk_6	0	0	0	1	0	10	1	94	86	81	3	0	120
Unk_7	0	0	0	0	0	29	0	159	32	3	2	0	171
Unk_8	0	0	1	0	0	0	0	1	0	0	9	0	95
Unk_9	0	0	0	0	0	0	0	0	0	0	0	0	17

Note: Clusters are labeled according to majority cell type, with the exception of clusters(*) where label is according to relevant cell types (highlighted in blue).

Supplementary Table 3: Antibodies used in flow cytometry panels

Antibody	Clone	Dilution	Manufacturer	Catalogue number
Arg1	A1exF5	1:200	eBioscience	12-3697-82
Calreticulin	EPR3924	1:500	Abcam	ab196159
CD4	RM4-5	1:200	BD Horizon	563151
CD8a	53-6.7	1:200	BioLegend	100706
CD11b	M1/70	1:200	BD Horizon	562950
CD11c	N418	1:100	eBioscience	11-0114-85
CD38	90	1:500	BioLegend	102718
CD44	IM7	1:500	BD Horizon	5663736
CD45.2	104	1:100	BioLegend	109824
CD47	miap301	1:200	eBioscience	25-0471-80
CD80	16-10A1	1:200	eBioscience	25-0801-82
CD86	GL1	1:200	BioLegend	105043
CD195 (CCR5)	4B12	1:200	BioLegend	107018
CD206	MR6F3	1:200	eBioscience	17-2061-82
F4/80	BM8	1:100	BioLegend	123132
FoxP3	FJK-16s	1:1000	eBioscience	45-5773-82
Granzyme B	GB11	1:100	BD Pharmingen	560213
I-A/I-E	M5/114	1:2000	BioLegend	107614
IFNγ	XMG1.2	1:200	BD Bioscience	563376
Ki67	SolA15	1:100	eBioscience	48-5698-82
Ly6C	HK1.4	1:1000	BioLegend	128012
Ly6G	1A8	1:1000	BD Pharmingen	551461
NOS2	CXNFT	1:500	eBioscience	46-5920-82
TNFα	MPG-XT22	1:500	BD Pharmingen	554419

Supplementary Table 4: Flow cytometry gating strategy for cell types

**Cell Type**	**Gating Strategy**
$CD8^+$ T Cells	Live>Singlets>LIVE/Dead Dye^{neg}>$CD45^+$>$CD11b^{lo}$>$CD8^+$
$CD4^+$ T cells	Live>Singlets>LIVE/Dead Dye^{neg}>$CD45^+$>$CD11b^{lo}$>$CD4^+$>$FoxP3^{neg}$
Tregs	Live>Singlets>LIVE/Dead Dye^{neg}>$CD45^+$>$CD11b^{lo}$>$CD4^+$>$FoxP3^+$
Macrophages	Live>Singlets>LIVE/Dead Dye^{neg}>$CD45^+$>$CD11b^{hi}$>$Ly6G^{neg}$>$F4/80^+$
PMN-MDSC/ Neutrophils	Live>Singlets>LIVE>Dead Dye^{neg}>$CD45^+$>$CD11b^{hi}$>$Ly6C^{med}$>$Ly6G^{hi}$
M-MDSC	Live>Singlets>LIVE/Dead Dye^{neg}>$CD45^+$>$CD11b^{hi}$>$Ly6G^{neg}$>$Ly6C^{hi}$
DC	Live>Singlets>LIVE/Dead Dye^{neg}>$CD45^+$>$Ly6G^{neg}$>$F4/80^{neg}$>$CD11c^+$
Non-immune/Tumor cells	Live>Singlets>LIVE/Dead Dye^{neg}>$CD45^{neg}$

DC, dendritic cells; M-MDSC, monocytic myeloid-derived suppressor cells; PMN-MDSC, polymorphonuclear myeloid-derived suppressor cells; Tregs, regulatory T cells

Supplementary Table 5: List of GO, KEGG, REACTOME, and HALLMARK pathways downregulated in all tumor-infiltrating neutrophils identified by scRNAseq upon CD8 depletion in mice treated with entinostat + NHS-IL12 as in Fig. 1a. Pathway highlighted in bold is shown in Supplementary Figure 7k.

Pathway Name	Category	Pathway Accession Identifier	pval
HALLMARK_INTERFERON_GAMMA_RESPONSE	H	M5913	0.033571443

Supplementary Methods

Histopathology. Brain, heart, lung, liver, kidney and small intestine were removed and stored in 10% formalin. Paraffin embedded sections were stained for hematoxylin and eosin (H&E) and pathology was determined by an independent certified pathologist (VitroVivo, Inc.).

NHS-rmIL12 labeling with Alexa Fluor 647 (A647) and *ex vivo* imaging. NHS-rmIL12 was conjugated to A647 using the SAIVI Alexa Fluor 647 Antibody/Protein 1 mg Labeling Kit from ThermoFisher Scientific following the manufacturer's instructions. UV-visible spectroscopy revealed the successful conjugation of 4.63 moles of fluorophore per mole of protein. The conjugation of A647 to NHS-rmIL12 was also confirmed by denaturing gel electrophoresis. The heavy and light chain of NHS and the rmIL12 were separated on 12% SDS-PAGE precast gels in 1× morpholinepropanesulfonic acid (MOPS) buffer for 40 min at 200 V and 120 mA in the presence of Chameleon duo protein size markers. Gels were stained with Coomassie Brilliant Blue (0.25% w/v) and subsequently imaged under white light and red fluorescent light. Following successful conjugation, EMT6 tumor-bearing Balb/c mice were subcutaneously injected with 50 μg of A647 labelled NHS-rmIL12. 24 hours later, mice were sacrificed, and tumors were extracted to be imaged using the Odyssey CLx (LI-COR). The level of NHS-rmIL12–generated fluorescence was quantified using ImageJ.

Generating and polarizing bone marrow derived macrophages (BMDM). The femurs and tibias of PBS-treated EMT6 tumor-bearing Balb/C mice were aseptically removed and cleaned. Bone marrow was flushed from the bones, washed, and cultured in petri dishes with 20ng/mL of M-CSF (Peprotech) for 6 days. On the 6th day, the BMDM were lifted off the dish with Accutase

(Innovative Cell Technologies) and replated with media containing only M-CSF (20ng/mL) for M0 or M-CSF + IFNγ (20ng/mL, Peprotech) + TNFα (20ng/mL, Peprotech) for M1 polarization. Twenty-four hours later the polarized BMDM were lifted with Accutase and the expression of the M1- and M2-like markers was examined by flow cytometry.

ScRNA-seq data analysis. Analysis of single cell data was performed with a standard workflow (Seurat v.3, 1), through a user interface developed on the Foundry Platform (Palantir Technologies) [1]. Raw data (GEO #GSE171273, https://www.ncbi.nlm.nih.gov/geo/query/acc.cgi?acc=GSE171273) was filtered using a mitochondrial content cutoff of 25%. Marker genes for specific cell types were obtained from the literature [2-4] and listed in Supplementary Table 1. A cell type score was calculated for each cell based on the AddModuleScore function as implemented in the Seurat package [5, 6] and cell identities determined using the following criteria: the module score for the cell type had to exceed a cell type-specific module score threshold and have the highest module score among all cell types. Cells that did not pass threshold scores for any cell type were labeled "unknown." Cell clusters were calculated using the Louvain algorithm with multilevel refinement [5]. Clusters were identified by the majority of cells represented in the cluster with the exception of M1- and M2-Macrophage clusters where the relevant cell types were highlighted for significant presence (Supplementary Table 2).

Clinical relevance of the murine studies. To confirm that the murine findings would correlate to benefits in patients, we analyzed data from the Cancer Genome Atlas (TCGA) using the open source platforms TIMER2.0 (http://timer.cistrome.org) and the Gene Expression Profiling Interactive Analysis (GEPIA2, http://gepia2.cancer-pku.cn/about.html), which are freely

available to all users. The RNA-Seq datasets GEPIA2 uses are based on the UCSC Xena project (http://xena.ucsc.edu), which are computed by a standard pipeline. The GEPIA2 project was supported by grants from Beijing Advanced Innovation Center for Genomics at Peking University, Key Technologies R&D Program (2016YFC0900100) and National Natural Science Foundation of China (81573022, 31530036). TIMER2.0 provides robust estimations of immune infiltration level for human data included in TCGA using six state-of-the-art algorithms, including TIMER, xCell, MCP-counter, CIBERSORT, EPIC, and quanTIseq. All TCGA tumor data present on TIMER2.0 were collected from the GDAC firehose website (http://firebrowse.org/). We used TIMER2.0 to correlate genes of interest from our murine studies with immune infiltrations in cancer patients in various human cancer subtypes. GEPIA2 is a newly developed online software, which is based on the sequencing database of 9736 tumor samples across 33 cancer types and 8587 normal samples from TCGA and GTEx programs [7]. We used GEPIA2 to explore the differential expression of genes of interest in various human cancer subtypes, and to correlate patient survival with the expression of a signature set of designated genes. To visualize the overall survival patterns with Kaplan-Meier curves, cohorts were subdivided using population medians, with a 50% cut-off (top 50% or low 50%), based on a 95% confidence interval. Hazard ratios were calculated based on Cox PH model.

Supplementary References

1. Chariou, P., et al. *CCBR/Antitumor-activity-of-entinostat-plus-NHS-IL12: manuscript methods. doi.org/10.5281/zenodo.4891285.* 2021.

2. Zilionis, R., et al., *Single-cell transcriptomics of human and mouse lung cancers reveals conserved myeloid populations across individuals and species.* Immunity, 2019. **50**(5): p. 1317-1334 e10.

3. Ponzetta, A., et al., *Neutrophils driving unconventional T cells mediate resistance against murine sarcomas and selected human tumors.* Cell, 2019. **178**(2): p. 346-360 e24.

4. Zhang, L., et al., *Single-Cell Analyses Inform Mechanisms of Myeloid-Targeted Therapies in Colon Cancer.* Cell, 2020. **181**(2): p. 442-459 e29.

5. Stuart, T., et al., *Comprehensive integration of single-cell data.* Cell, 2019. **177**(7): p. 1888-1902 e21.

6. Tirosh, I., et al., *Dissecting the multicellular ecosystem of metastatic melanoma by single-cell RNA-seq.* Science, 2016. **352**(6282): p. 189-96.

7. Tang, Z., et al., *GEPIA2: an enhanced web server for large-scale expression profiling and interactive analysis.* Nucleic Acids Res, 2019. **47**(W1): p. W556-W560.
