# Supplementary material for: Tumour-targeted interleukin-12 and entinostat combination therapy improves cancer survival by reprogramming the tumour immune cell landscape
Source: Nat Commun. 2021 Aug 26;12:5151. doi: 10.1038/s41467-021-25393-x (PMC8390765; doi:10.1038/s41467-021-25393-x)
Supplement: Supplementary file 2 — Description of Additional Supplementary Files [file 41467_2021_25393_MOESM2_ESM.pdf]

**Description of Additional Supplementary Files**

File Name: Supplementary Data 1
Description: List of GO, KEGG, REACTOME, and HALLMARK pathways upregulated in CD8+ tumor-infiltrating lymphocytes by scRNAseq in mice treated with entinostat + NHS-IL12 versus PBS-treated control tumors as in Fig. 1a on day 21 post-tumor implant. Pathways highlighted in bold are shown in Fig. 4a.

File Name: Supplementary Data 2
Description: List of GO, KEGG, REACTOME, and HALLMARK pathways upregulated in tumor-infiltrating neutrophils identified by scRNAseq in mice treated with entinostat + NHS-IL12 versus PBS-treated control tumors as in Fig. 1a on day 21 post-tumor implant. Pathways highlighted in bold are shown in Fig. 5f.

File Name: Supplementary Data 3
Description: List of GO, KEGG, REACTOME, and HALLMARK pathways upregulated in all tumor-infiltrating monocytes identified by scRNAseq in mice treated with entinostat + NHS-IL12 treated as in Fig. 1a versus PBS-treated control tumors on day 21 post-tumor implant. Pathways highlighted in bold are shown in Supplementary Fig. 5a.

File Name: Supplementary Data 4
Description: List of GO, KEGG, REACTOME, and HALLMARK pathways upregulated in tumor-infiltrating macrophages not identified as M1 or M2 by scRNAseq in mice treated with entinostat + NHS-IL12 versus PBS-treated control tumors as in Fig. 1a on day 21 post-tumor implant. Pathways highlighted in bold are shown in Fig. 6b.

File Name: Supplementary Data 5
Description: List of GO, KEGG, REACTOME, and HALLMARK pathways upregulated in tumor-infiltrating macrophages identified as M1 by scRNAseq in mice treated with entinostat + NHS-IL12 versus PBS-treated control tumors as in Fig. 1a on day 21 post-tumor implant. Pathways highlighted in bold are shown in Fig. 6d.

File Name: Supplementary Data 6
Description: List of GO, KEGG, REACTOME, and HALLMARK pathways downregulated in all tumor-infiltrating macrophages identified by scRNAseq in CD8-depleted mice treated with entinostat + NHS-IL12 as in Fig. 1a versus undepleted. Pathways highlighted in bold are shown in Fig. 7d.
