# Supplementary material for: Tumour-targeted interleukin-12 and entinostat combination therapy improves cancer survival by reprogramming the tumour immune cell landscape
Source: Nat Commun. 2021 Aug 26;12:5151. doi: 10.1038/s41467-021-25393-x (PMC8390765; doi:10.1038/s41467-021-25393-x)
Supplement: Supplementary file 9 — Reporting Summary [file 41467_2021_25393_MOESM9_ESM.pdf]

nature research

Corresponding author(s): Jeffrey Schlom

Last updated by author(s): 06/16/2021

Reporting Summary

Nature Research wishes to improve the reproducibility of the work that we publish. This form provides structure for consistency and transparency in reporting. For further information on Nature Research policies, see our Editorial Policies and the Editorial Policy Checklist.

Statistics

For all statistical analyses, confirm that the following items are present in the figure legend, table legend, main text, or Methods section.

n/a	Confirmed	
☐	☒	The exact sample size (*n*) for each experimental group/condition, given as a discrete number and unit of measurement
☐	☒	A statement on whether measurements were taken from distinct samples or whether the same sample was measured repeatedly
☐	☒	The statistical test(s) used AND whether they are one- or two-sided<br>*Only common tests should be described solely by name; describe more complex techniques in the Methods section.*
☒	☐	A description of all covariates tested
☒	☐	A description of any assumptions or corrections, such as tests of normality and adjustment for multiple comparisons
☐	☒	A full description of the statistical parameters including central tendency (e.g. means) or other basic estimates (e.g. regression coefficient) AND variation (e.g. standard deviation) or associated estimates of uncertainty (e.g. confidence intervals)
☐	☒	For null hypothesis testing, the test statistic (e.g. F, t, r) with confidence intervals, effect sizes, degrees of freedom and P value noted<br>*Give P values as exact values whenever suitable.*
☒	☐	For Bayesian analysis, information on the choice of priors and Markov chain Monte Carlo settings
☒	☐	For hierarchical and complex designs, identification of the appropriate level for tests and full reporting of outcomes
☐	☒	Estimates of effect sizes (e.g. Cohen's *d*, Pearson's *r*), indicating how they were calculated

Our web collection on statistics for biologists contains articles on many of the points above.

Software and code

Policy information about availability of computer code

Data collection	BD FACS Diva v 9.0 software, BD FACSuite v1.0.6.5320, 10X Genomics cellranger v 4.0.0, ImmunoSpot SC Suite v 2.6.1; Odyssey CLx, Zen Blue v 2.
Data analysis	GraphPad Prism v 7.0; ImmunoSpot SC Suite v 2.6.1; FlowJo v 9.9.6; 10X Genomics cellranger v 4.0.0; Seurat v.3.1. with Louvain algorithm with multilevel refinement; TIMER2.0, GEPIA 2.<br>All scRNASeq data were analyzed using custom code written in R v.4.0.1 based on open source software. All scripts used have been deposited in github: https://github.com/CCBR/Antitumor-activity-of-entinostat-plus-NHS-IL12. DOI: doi.org/10.5281/zenodo.4891285

For manuscripts utilizing custom algorithms or software that are central to the research but not yet described in published literature, software must be made available to editors and reviewers. We strongly encourage code deposition in a community repository (e.g. GitHub). See the Nature Research guidelines for submitting code & software for further information.

Data

Policy information about availability of data

All manuscripts must include a data availability statement. This statement should provide the following information, where applicable:

- Accession codes, unique identifiers, or web links for publicly available datasets
- A list of figures that have associated raw data
- A description of any restrictions on data availability

ScRNASeq data has been deposited in GEO, #GSE171273 (https://www.ncbi.nlm.nih.gov/geo/query/acc.cgi?acc=GSE171273). Human gene transcript TCGA (https://portal.gdc.cancer.gov) and GTEx (http://gtexportal.org) datasets are identified in figure legends and were accessed through TIMER2.0 (http://timer.comp-genomics.org) and/or GEPIA2 (http://gepia2.cancer-pku.cn). Additional source data are provided with this paper. All relevant data are available from the authors upon request.

Field-specific reporting

Please select the one below that is the best fit for your research. If you are not sure, read the appropriate sections before making your selection.

☒ Life sciences ☐ Behavioural & social sciences ☐ Ecological, evolutionary & environmental sciences

For a reference copy of the document with all sections, see nature.com/documents/nr-reporting-summary-flat.pdf

Life sciences study design

All studies must disclose on these points even when the disclosure is negative.

Sample size	In all animal studies, a minimum of 6 animals/group were used for analysis of immune correlates, and a minimum of 7 animals/group were used for anti-tumor and survival studies. No power analysis was performed as these tumor models are well established and yield low variance. Owing to this low variance, statistical power is usually achieved with 5-6 animals per group. To account for potential misinjection of tumor cells or lack of tumor engraftment, at least 6 mice/group were approved to be used in these experiments. The number of animals per group for tumor re-challenge studies were based on the number of cured mice to be re-challenged, with a minimum of 5. All other experimental groups in this study contained at least 6 animals.
Data exclusions	We did not exclude individual results from the presented data.
Replication	Anti-tumor studies were performed 3 times (EMT6), twice (CT26, nu/nu) and once (MC38). All EMT6 immune studies were performed 2-3 times, except for data on day 17 post-tumor implant, which was performed once. All EMT6 and CT26 replicate experiments were successful and yielded similar results. MC38 immune data was collected once. MC38 anti-tumor and immune studies were not replicated due to COVID-19 restrictive laboratory work. Single-cell RNAseq was performed once and not repeated due to COVID-19 restrictive laboratory work.
Randomization	Individual animals were randomized based on tumor size into two treatment cohorts (control and entinostat chow) at the time of initiation of entinostat treatment (50-100mm3). A second randomization was performed in each cohort when control tumors reached a mean size of 150-200mm3, prior to initiation of NHS-IL12 treatment.
Blinding	Non-blinding experiments. Tumor implants in all in vivo studies were performed independently in a non-blind fashion by two different investigators and data was always collected blindly by technical staff. Ex vivo studies were performed every time by a team effort involving multiple investigators, including ELISPOT, flow cytometry, cytokine and chemokine analysis, etc. In all ex-vivo studies, sample preparation, staining, data acquisition and analysis were performed with all study samples batched. Sera preparation was performed non-blindly but cytokine data acquisition and analysis were performed blindly. scRNAseq sample preparation was performed in a non-blind fashion but data acquisition and analysis were performed blindly. All studies, including data collection and analysis, were closely monitored.

Reporting for specific materials, systems and methods

We require information from authors about some types of materials, experimental systems and methods used in many studies. Here, indicate whether each material, system or method listed is relevant to your study. If you are not sure if a list item applies to your research, read the appropriate section before selecting a response.

Materials & experimental systems

n/a	Involved in the study	
☐	☒	Antibodies
☐	☒	Eukaryotic cell lines
☒	☐	Palaeontology and archaeology
☐	☒	Animals and other organisms
☒	☐	Human research participants
☒	☐	Clinical data
☒	☐	Dual use research of concern

Methods

n/a	Involved in the study	
☒	☐	ChIP-seq
☐	☒	Flow cytometry
☒	☐	MRI-based neuroimaging

Antibodies

Antibodies used

We used the following mouse specific antibodies in our studies:
'Antigen', 'color', 'clone', 'supplier', 'catalog number', 'lot number', 'dilution, 'isotype'
- Arg1: PE, eBioscience, clone A1exF5, REF 12-3697-82, lot 2054345, 1:200, Rat IgG2a,k
- Calreticulin: AF647, clone EPR3924, Abcam, REF ab196159, lot GR3290257-2, 1:500, IgG
- CD4: BV605, clone RM4-5, BD Horizon, REF 563151, lot 0314025, 1:200, Rat IgG2a,k
- CD8a: FITC, clone 53-6.7, BioLegend, REF 100706, lot B195504, 1:200, Rat IgG2a,k
- CD11b: BV510, clone M1/70, BD Horizon, REF 562950, lot 0209403, 1:200, Rat IgG2b,k
- CD11c: FITC, clone N418, eBioscience, REF 11-0114-85, lot E00155-1632, 1:100, Armenian Hamster IgG
- CD38: PECy7, clone 90, BioLegend, REF 102718, lot B288231, 1:500, Rat IgG2a,k
- CD44: BV786, clone IM7, BD Horizon, REF 5663736, lot 9142890, 1:500, Rat IgG2b,k

- CD45.2: APC Cy7, clone 104, BioLegend, REF 109824, lot B294042, 1:100, Mouse IgG2a,k
- CD47: PE-Cy7, clone miap301, eBioscience, REF 25-0471-80, lot 2138815, 1:200, Rat IgG2a,k
- CD80: PECy7, clone 16-10A1, eBioscience, REF 25-0801-82, lot 4303332, 1:200, Armenian Hamster IgG
- CD86: BV786, clone GL1, BioLegend, REF 105043, lot B250421, 1:200, Rat IgG2a,k
- CD195: PE-Cy7, clone 4B12, BioLegend, REF 107018, lot B263926, 1:200, Armenian Hamster IgG
- CD206: APC, clone MR6F3, eBioscience, REF 17-2061-82, lot 2140730, 1:200, Rat IgG2a,k
- F4/80: BV421, clone BM8, BioLegend, REF 123132, lot B265669, 1:100, Rat IgG2a,k
- FoxP3: PerCP-Cy5.5, clone FJK-16s, eBioscience, REF 45-5773-82, lot 2005226, 1:1000, Rat IgG2a,k
- Granzyme B: AF700, clone GB11, BD Pharmingen, REF 560213, lot 9002854, 1:100, Mouse IgG1,k
- I-A/I-E: APC, clone M5/114, BioLegend, REF 107614, lot B191785, 1:2000, Rat IgG2b,k
- IFNgamma: BV421, clone XMG1.2, BD Bioscience, REF 563376, lot 0205737, 1:200, Rat IgG1,k
- Ki67: e450, clone SolA15, eBioscience, REF 48-5698-82, lot 2082927, 1:100, Rat IgG2a,k
- Ly6C: PerCP-Cy5.5, clone HK1.4, BioLegend, REF 128012, lot B250462, 1:1000, Rat IgG2c,k
- Ly6G: PE, clone 1A8, BD Pharmingen, REF 551461, lot 0065521, 1:1000, Rat IgG2a,k
- NOS2: PerCP-Cy5.5, clone CXNFT, eBioscience, REF 46-5920-82, lot 4342191, 1:500, Rat IgG2a,k
- TNFalpha: PE, clone MPG-XT22, BD Pharmingen, REF 554419, lot 7271688, 1:500, Rat IgG1,k

For CD8 depletion, anti-CD8 (clone 2.43) from BioXCell (cat. #BP0061, lot #624616A2) was used.

Validation

All antibodies used in this study were validated by the manufacturers for specific detection of the antigen and species reactivity:
- Anti-mouse Arg1 was validated for flow cytometry in mouse bone marrow derived macrophages polarized with either IL-4 or LPS and IFN-gamma, and subsequently surface stained with F4/80 (clone BM8). Cells were then fixed and permeabilized, followed by intracellular staining with Arginase 1 (clone A1exF5).
- Anti-mouse Calreticulin was validated for flow cytometry using HAP1 wildtype and HAP1-CALR knockout cells stained with ab196159. A rabbit monoclonal IgG isotype control antibody was used at the same concentration and conditions as the primary antibody. Unlabelled sample was also used as a control.
- Anti-mouse CD4 was validated for flow cytometry using mouse splenocytes preincubated with purified Rat Anti-Mouse CD16/CD32 antibody, following by staining with BD Horizon BV605 Rat Anti-Mouse CD4 and APC Hamster Anti-Mouse CD3e antibodies.
- Anti-mouse CD8a was validated for flow cytometry using mouse splenocytes stained with anti-CD8 (clone 53-6.7) FITC or rat IgG2a, κ FITC isotype control.
- Anti-mouse CD11b was validated for flow cytometry using mouse bone-marrow cells stained with either BD Horizon BV510 Rat IgG2b, κ Isotype Control or BD Horizon BV510 Rat Anti-Mouse CD11b antibody.
- Anti-mouse CD11c was validated for flow cytometry using mouse splenocytes stained with CD11c-FITC, APC anti-mouse I-A/I-E (clone M5/114.15.2) and FITC N418 or FITC Armenian hamster IgG isotype control.
- Anti-mouse CD38 was validated for flow cytometry using mouse splenocytes stained with clone 90 PE-Cyanine7
- Anti-mouse CD44 was validated for flow cytometry using mouse bone-marrow cells preincubated with Purified Rat Anti-Mouse CD16/CD32 antibody, followed by staining with either BD Horizon BV786 Rat IgG2b, κ Isotype Control or BD Horizon BV786 Rat Anti-Mouse CD44 antibody.
- Anti-mouse CD45.2 was validated for flow cytometry using C57BL/6 splenocytes stained with CD45.2 (clone 104) APC-Cyanine7 or Mouse IgG2a, k APC-Cyanine7 isotype control.
- Anti-mouse CD47 was validated for flow cytometry using mouse splenocytes with Rat IgG2a K Isotype Control PE-Cyanine7 or Anti-Mouse CD47 PE-Cyanine7.
- Anti-mouse CD80 was validated for flow cytometry using unstimulated LPS-stimulated mouse splenocytes with Armenian Hamster IgG Isotype Control PE-Cyanine7 or Anti-Mouse CD80 (B7-1) PE-Cyanine7.
- Anti-mouse CD86 was validated for flow cytometry using LPS-stimulated mouse splenocytes stained with CD86 (clone GL-1) Brilliant Violet 785™ or rat IgG2a, κ Brilliant Violet 785™ isotype control.
- Anti-mouse CD195 was validated for flow cytometry using mouse CD195 (CCR5) transfected NRK cells stained with CD195 (clone HM-CCR5) PE/Cyanine7 or Armenian Hamster IgG PE/Cyanine7 isotype control.
- Anti-mouse CD206 was validated for flow cytometry using mouse resident peritoneal exudate cells surface stained with Anti-Mouse F4/80 Antigen eFluor® 450 followed by fixation, permeabilization, and intracellular stain with Rat IgG2b K Isotype Control APC or Anti-Mouse CD206 (MMR) APC.
- Anti-mouse F4/80 was validated for flow cytometry using thioglycolate-elicited mouse peritoneal macrophages stained with F4/80 (clone BM8) Brilliant Violet 421™ or rat IgG2a, κ Brilliant Violet 421™ isotype control.
- Anti-mouse FoxP3 was validated for flow cytometry using surface staining of mouse splenocytes with Anti-Mouse CD4 FITC followed by intracellular staining with Rat IgG2a K Isotype Control PerCP-Cyanine5-5 or Anti-Mouse/Rat Foxp3 PerCP-Cyanine5-5. Cells in the lymphocyte gate were used for analysis.
- Anti-mouse I-A/I-E was validated for flow cytometry using mouse splenocytes stained with anti-mouse I-A/I-E (clone M5/114.15.2)-APC or rat IgG2b, κ APC isotype control.
- Anti-mouse IFNg was validated for flow cytometry using phorbol/ionomycin-stimulated mouse splenocytes in the presence of BD GolgiStop™ Protein Transport Inhibitor (containing Monensin). Cells were harvested, washed, fixed, permeabilized, and stained with PE Rat Anti-Mouse CD4 antibody and either BD Horizon™ BV421 Rat IgG1, κ Isotype Control or BD Horizon™ BV421 Rat Anti-Mouse IFN-γ antibody.
- Anti-mouse Ki67 was validated for flow cytometry in mouse immune and non-immune cells using matched isotype control.
- Anti-mouse Ly6C was validated for flow cytometry using mouse bone marrow cells stained with HK1.4 PerCP/Cyanine5.5.
- Anti-mouse Ly6G was validated for flow cytometry using mouse bone-marrow cell suspensions stained with either PE Rat Anti-Mouse Ly-6G or PE Rat IgG2a, κ Isotype Control, in the presence of purified anti-mouse CD16/CD32. Non-viable leukocytes were excluded by staining with Propidium Iodide Staining Solution, and leukocyte subsets were distinguished by their light-scatter profiles.
- Anti-mouse NOS2 was validated for flow cytometry using unstimulated or LPS-stimulated mouse thioglycolate-elicited peritoneal exudate cells surface stained with Anti-Mouse F4/80 FITC. Cells were fixed and permeabilized then intracellularly stained with Anti-Mouse NOS2 PerCP-eFluor® 710.
- Anti-mouse TNFa was validated for flow cytometry in mouse immune cells.

Additionally, all antibodies were validated in an internal quality control process using lymphocytes recovered from congenic animals. All antibodies stained specifically the expected populations.

Anti-mouse CD8alpha (2.43) antigen specificity was validated by the manufacturer for specific detection of the murine CD8alpha antigen by immunoblot in mouse CTL clone L3 cells. This antibody is described by the manufacturer as exhibiting CD8 cell depleting activity when used in vivo. This antibody is widely used in publications [Immunity 48(4): 773-786 e775; Nat Med. doi: 10.1038/nm.4200; Nature 522(7556): 345-348] for the depletion of CD8+ T cells in mice. This was validated in our laboratory in this study in blood and tumor (Fig. 7g) and previous in vivo studies (ex: DOI: 10.1158/1078-0432.CCR-19-0727; doi: 10.1186/s40425-019-0551-y) in syngeneic mice.

Eukaryotic cell lines

Policy information about cell lines

Cell line source(s)	EMT6 and CT26 were obtained from ATCC (Manassas, VA, USA). TS/A were a kind gift from Dr. Donald Buchsbaum (University of Alabama at Birmingham) with permission from Dr. Pier-Luigi Lollini (Univ. Bologna, Italy) who was one of the investigators who established the cell line (DOI: 10.1007/BF00121199). MC38 were developed in-house.
Authentication	EMT6 and CT26 cell lines were obtained with the certificate from the vendor. TS/A and MC38 cell lines were not authenticated.
Mycoplasma contamination	All cell lines tested negative for Mycoplasma contamination.
Commonly misidentified lines (See ICLAC register)	None used.

Animals and other organisms

Policy information about studies involving animals; ARRIVE guidelines recommended for reporting animal research

Laboratory animals	Six- to 8-week-old female Balb/c, nu/nu and CEA-Tg mice (Mus musculus) were obtained from the NCI Frederick Cancer Research Facility (Frederick, MD).
Wild animals	None used.
Field-collected samples	None used.
Ethics oversight	Animals were housed in microisolator cages under pathogen-free conditions and a 12h:12h light/dark cycle, in rooms at 72°F±2°F and 30-70% relative humidity, in an Association for Assessment and Accreditation of Laboratory Animal Care-accredited animal facility of the National Institutes of Health (NIH) . All studies were reviewed and approved by the NIH Intitutional Animal Care and Use Committee. We have complied with all relevant ethical regulations for animal testing and research.

Note that full information on the approval of the study protocol must also be provided in the manuscript.

Flow Cytometry

Plots

Confirm that:

☒ The axis labels state the marker and fluorochrome used (e.g. CD4-FITC).

☒ The axis scales are clearly visible. Include numbers along axes only for bottom left plot of group (a 'group' is an analysis of identical markers).

☒ All plots are contour plots with outliers or pseudocolor plots.

☒ A numerical value for number of cells or percentage (with statistics) is provided.

Methodology

Sample preparation	Single-cell suspensions from tumor and spleen were prepared using standard procedures. Cell counts were performed using 123 count eBeads (ThermoFisher). Staining of immune cells (~1x10^6) for flow cytometry was performed using the Cytofix/Cytoperm Kit (BD Biosciences) according to the manufacturer's instructions. Antibodies used are listed in Supplementary Table 1 with matched isotypes obtained from the aforementioned manufacturers. Data were acquired on a BD FACSVerse, or LSRII Fortessa flow cytometer with FACS Diva v 9.0 software (BD Biosciences) and analyzed with FlowJo Analysis Software (BD Biosciences). Cell populations were identified using gating strategies shown in Supplementary Table 2 and Supplementary Figure 9. All frequencies of phenotypic proteins were generated by subtracting the frequency of respective isotype, typically set between 1-5%.
Instrument	LSRII Fortessa
Software	Data was acquired on a BD FACS Diva v 9.0 and analyzed using FlowJo v 9.0.
Cell population abundance	No cell sorting was performed in these studies.
Gating strategy	We used standard gating strategies: Live/Dead discrimination based on FSC and SSC signals, exclusion of doublets using FSC-A

Gating strategy

and FSC-H, gating on Live cells negative for LIVE/DEAD dye, following by specific gating for each cell type as described in the submitted Supplementary File, Table 2 and Supplementary Figure 9.

☒ Tick this box to confirm that a figure exemplifying the gating strategy is provided in the Supplementary Information.
